# Androgen Receptor Functional Analyses by High Throughput Imaging: Determination of Ligand, Cell Cycle, and Mutation-Specific Effects

**DOI:** 10.1371/journal.pone.0003605

**Published:** 2008-11-03

**Authors:** Adam T. Szafran, Maria Szwarc, Marco Marcelli, Michael A. Mancini

**Affiliations:** 1 Departments of Molecular and Cellular Biology and Medicine, Baylor College of Medicine, Houston, Texas, United States of America; 2 The Michael E. DeBakey VA Medical Center, Baylor College of Medicine, Houston, Texas, United States of America; Institute of Genetics and Molecular and Cellular Biology, France

## Abstract

**Background:**

Understanding how androgen receptor (AR) function is modulated by exposure to steroids, growth factors or small molecules can have important mechanistic implications for AR-related disease therapies (e.g., prostate cancer, androgen insensitivity syndrome, AIS), and in the analysis of environmental endocrine disruptors.

**Methodology/Principal Findings:**

We report the development of a high throughput (HT) image-based assay that quantifies AR subcellular and subnuclear distribution, and transcriptional reporter gene activity on a cell-by-cell basis. Furthermore, simultaneous analysis of DNA content allowed determination of cell cycle position and permitted the analysis of cell cycle dependent changes in AR function in unsynchronized cell populations. Assay quality for EC50 coefficients of variation were 5–24%, with Z' values reaching 0.91. This was achieved by the selective analysis of cells expressing physiological levels of AR, important because minor over-expression resulted in elevated nuclear speckling and decreased transcriptional reporter gene activity. A small screen of AR-binding ligands, including known agonists, antagonists, and endocrine disruptors, demonstrated that nuclear translocation and nuclear “speckling” were linked with transcriptional output, and specific ligands were noted to differentially affect measurements for wild type versus mutant AR, suggesting differing mechanisms of action. HT imaging of patient-derived AIS mutations demonstrated a proof-of-principle personalized medicine approach to rapidly identify ligands capable of restoring multiple AR functions.

**Conclusions/Significance:**

HT imaging-based multiplex screening will provide a rapid, systems-level analysis of compounds/RNAi that may differentially affect wild type AR or clinically relevant AR mutations.

## Introduction

The androgen receptor (AR), a member of the nuclear receptor superfamily, functions to regulate gene expression in response to androgens such as testosterone (T) and dihydrotestosterone (DHT). Several cell-based imaging models have been generated in recent years to study AR action, enabling researchers to correlate transcriptional competence of AR with some obligatory intracellular steps visible by fluorescence microscopy. These steps fit within the classical model of AR function: in response to ligands, AR sheds heat shock proteins, forms dimers, and translocates into the nucleus [1]–[3]. Upon entering the nucleus, AR then organizes into thousands of discrete but unstable foci (referred to as the hyperspeckled pattern), interacts with coregulators and members of the general transcriptional apparatus, and regulates gene expression by interacting with androgen response elements associated with androgen-regulated genes. The microscopic model of antagonist-treated-AR has similarities, such as induction of nuclear translocation, and differences, including a diminished hyperspeckled pattern and repressed transcription function [3].

AR signaling leads to differentiation of the male sexual phenotype, and maturation of the secondary sex characteristics, as well to maintenance of male libido, muscle mass and bone density. Disruption of this signaling through inactivating mutations of AR can lead to androgen insensitivity syndromes (AIS), in which genotypic males are affected by a spectrum of developmental abnormalities of the genital apparatus and of the secondary sexual characteristics [4], [5]. In addition to its role in AIS, AR is important in prostate cell proliferation, differentiation and survival, and plays at least a permissive role in development of prostate cancer [6]. Current therapy for advanced prostate cancer targets AR through the use of LHRH agonists and/or anti-androgens such as hydroxyflutamide or bicalutamide (Casodex). These drugs work by inhibiting androgen synthesis, or by preventing endogenous androgens from activating AR, respectively. While these treatments are initially successful, patients will eventually relapse in 18–24 months and present with androgen depletion-independent (ADI) disease, for which there is no effective cure; consequently, ADI results in approximately 30,000 deaths per year in the United States [7]. The molecular basis of transition to ADI is still incompletely characterized, however several androgen receptor-based hypotheses have been formulated [8], and they share the common denominator that AR acquires the ability to signal even in the androgen-depleted or AR-inhibited environment [9]. Some of the AR-based hypotheses to explain the development of ADI disease include development of activating AR mutations [10], AR activation by testosterone and dihydrotestosterone, which can be present in recurrent prostate cancer tissue at levels sufficient to stimulate AR [11], AR activation by a pool of ligands generated intraprostatically by increased expression of genes regulating androgen metabolism [12], or even AR activation by anti-androgens [13].

Some AR functions can now be investigated using automated single cell microscopy [3], [14]. This novel technology can be used to investigate unanswered questions related to AR physiopathology and to facilitate novel approaches to drug discovery. For instance, there is the need to examine at the single cell level how AR function is affected by various compounds, including traditional AR agonists and antagonists, precursors of testosterone, steroidal and non-steroidal substances known to bind AR with high or low affinity, and how these ligand receptor interactions are affected by AR mutations found in AIS and prostate cancer. In addition, due to the fact that AR plays a major role in the embryologic development of the male sexual phenotype and in spermatogenesis, there exist concerns on whether exposure to environmental compounds that disrupt normal endocrine pathways may affect AR-regulated functions [15]. Inasmuch that endocrine disruptors are increasingly being identified in the environment at bioactive levels and we do not know to what degree they affect AR function, there is the need to thoroughly study them at a single cell, system biology level to understand their mechanism of action.

Regarding drug discovery programs to identify the next generation of AR agonists or antagonists, a number of cell-based assays have been developed in the past, and most are based on use of a luciferase reporter gene. The reporter gene is placed under control of an AR-promoter or, in composite systems using Gal4-AR fusions, a UAS- promoter, either transiently or stably transfected into a selected cell lines. There are several limitations to this approach. **First**, the results are intrinsically based on cumulative data derived from thousands/millions of cells that certainly vary in terms of cell cycle and/or AR expression level (either endogenous or transiently/stably introduced). These are both important shortcomings, as changes in the amount of expression of a transcription factor affect its intracellular mobility [3], ability to interact with members of the transcriptional apparatus and to transcribe target genes [16], and cell cycle dependent sensitivity to AR functions have been suggested (see below; [17]). **Second**, luciferase assays provide single read-outs, yielding information only on the transcriptional reporter gene activity of AR, and are unable to contribute information on how AR cellular distribution, subnuclear organization and mobility, promoter occupancy and chromatin modeling are affected by various compounds. A single cell-based multiplex assay would have the ability to overcome some of these shortcomings, and more directly provide information on the mechanism of action of novel AR agonists or antagonists.

The importance of analyzing receptor activity at the single cell level is highlighted by the role of cell cycle in nuclear receptor transcriptional reporter gene activity. Early studies of the glucocorticoid receptor indicated reduced receptor activity in G2 [18]. A more recent study examining PR in T47D cells found highest PR activity in S phase. This activity was associated with increased nuclear localization of the receptor and elevated interaction with the nuclear receptor coactivators SRC-1 and SRC-3 [17]. For AR, earlier studies have indicated that AR has reduced activity in cells blocked in late G1/S phase compared to G0 or S-phase [19]. Few of these studies have examined the mechanisms behind the altered activity, and all have relied upon an external agent to enrich cell populations in a particular phase of the cell cycle. A single cell assay using selected markers would provide the ability to examine the effects of cell cycle on the basic mechanism of AR signaling without resorting to toxic inhibitors that can, at best, only partially synchronize cells.

Here, we report the development and utilization of a novel high throughput image-based transcriptional assay to study multiple aspects of AR intracellular biology at the single cell level. The assay is based upon image acquisition using robotic fluorescent microscopy and automated image analysis, generally referred to as high content screening (HCS) [20]. We have expanded upon our previous HCS efforts by using androgen-responsive HeLa cell lines that stably express either wild type or mutant AR fused to a green fluorescent protein in combination with a probasin promoter-based transcriptional reporter gene. This allows us to simultaneously quantify changes in AR nuclear translocation, nuclear patterning, and transcriptional reporter gene activity in response to compounds and AR mutations. Incorporation of EdU, a BrdU-like marker for newly synthesized DNA also allows for the cell cycle analyses in unsynchronized cell populations. We demonstrate responses to a small panel of ligands and examine the importance of AR expression level, the link between AR nuclear patterning and transcriptional reporter gene activity, the relationship between observed responses and cell cycle, and the functional impact of the LNCaP T877A and AIS F764L mutations.

## Results

### Assay System

**Figure 1:**
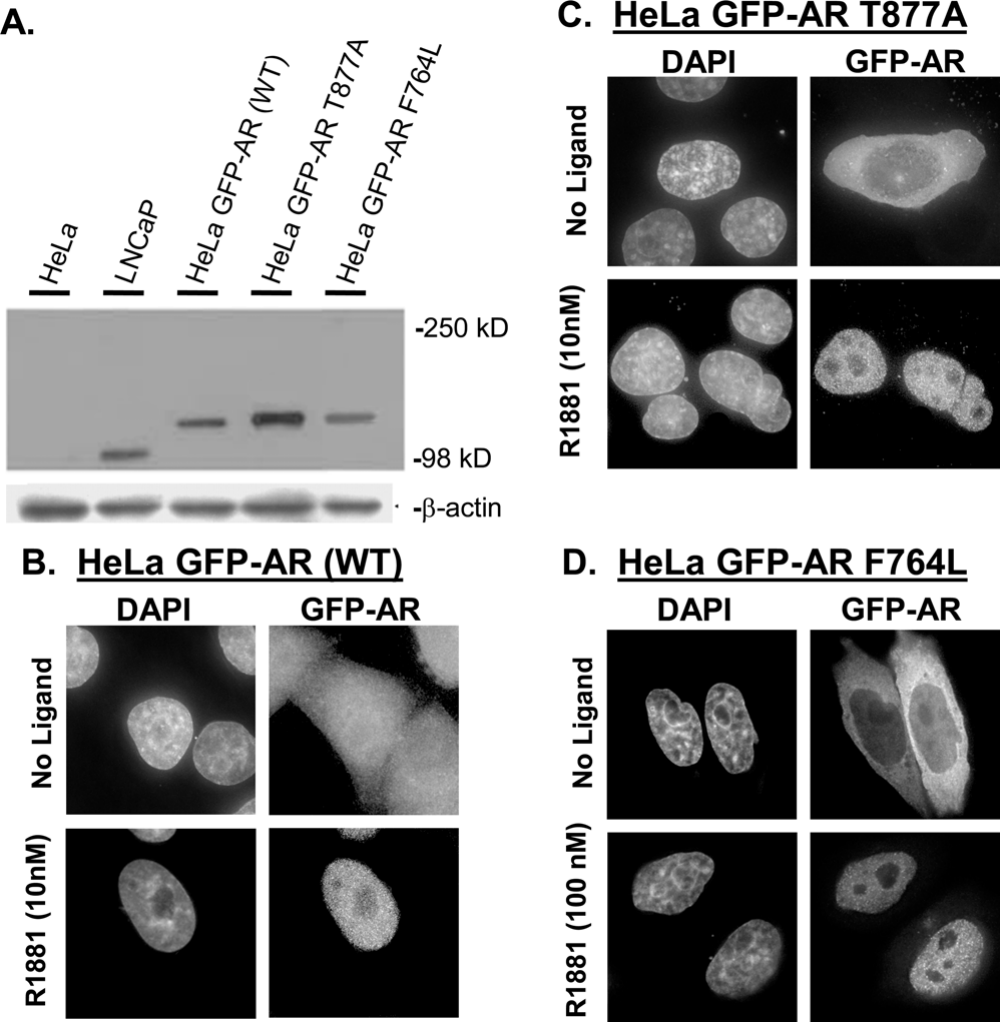
HeLa GFP-AR cell lines expressing wild type and mutant AR able to translocate into nucleus in response to agonist. A. Western blot analysis comparing HeLa (1), LNCaP (2), HeLa GFP-AR (3), HeLa GFP-AR T877A (4), and HeLa GFP-AR F764L (5) androgen receptor expression. Equal total protein levels were loaded for all cell abstracts and confirmed with β-actin control. B, C, and D. Deconvolution images of HeLa cell lines expressing stably integrated GFP-AR, GFP- AR T877A (LNCaP, ligand binding domain mutation), and GFP- AR T877A (AIS associated mutation, ligand binding domain mutation), shown without (top) and with 10 nM or 100 nM R1881 (bottom). The LBD mutation in GFP-AR T877A or GFP-F764L does not affect the ability to translocate into the nucleus.

To examine how wild-type and mutant ARs (expressed at physiologically relevant levels) respond to various experimental manipulations, HeLa cell lines were generated that stably express either wild type (GFP-AR), mutant GFP-ART877A (LNCaP mutation; [13]), or GFP-ARF764L (AIS mutation; [21]) under control of the CMV promoter. The T877A and F764L mutations were selected due to known altered ligand responses [13]. Generated cell lines were characterized at the population level by western blot analysis which indicated that HeLa GFP-AR, HeLa GFP-AR T877A, and HeLa GFP-AR F764L expressed AR of the expected size and at levels approximately 1.1-, 2.1, and 0.8-fold of that found in LNCaP cell pools (Fig. 1A). Furthermore, microarray-based RNA expression analysis demonstrated that GFP-AR regulates (activation or repression) known endogenous AR-responsive genes in response to ligand, indicating the cellular machinery of HeLa readily supports AR transcription function (Supplementary Table S1).

**Figure 2:**
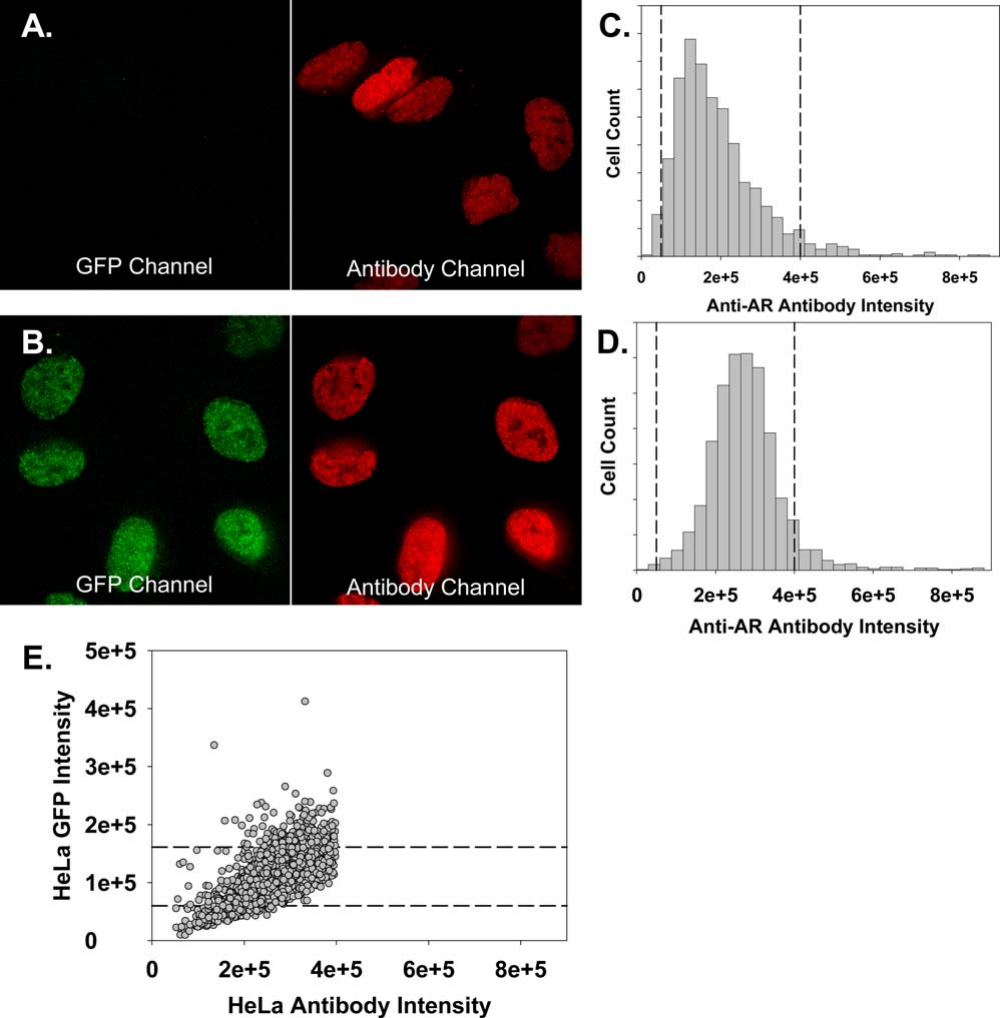
HTM analysis allows selection of cells expressing physiologically relevant levels of GFP-AR. A and B. Representative images of LNCaP cells (A) and HeLa cells stably transfected with GFP-AR (B) were immunolabeled and imaged to determine the GFP-AR signal that corresponds to endogenous LNCaP AR expression levels. Exposure levels for the GFP (left) and anti-AR labeling (right) is identical for both cell types. C. Image analysis was performed on the LNCaP antibody images to quantify the total anti-AR labeling per cell, derived from the standard 10×10 field of cells, containing >500 cells. The dashed lines indicate the 10% and 90% percentile of the population. D. Similar image analysis was performed on the HeLa GFP-AR antibody images to quantify the total anti-AR labeling per cell. The dashed lines indicate the 10%–90% expression range found in the LNCaP cell line. These cut-offs were empirically determined to remove the outliers, either barely-detectable GFP, or grossly over-expressing cells. As can be seen in the histogram from the ∼500 cell quantitation, removing the very heterogenous top 10%, or the more homogeneous bottom 10%, only eliminates the extremes, which can have an untoward influence on the bulk of the population. E. HeLa GFP-AR cells with AR expression within this range were selected and total GFP intensity per cell was determined and plotted. GFP expression corresponding to the 10% and 90% percentile in this population were determined (dashed lines) and used as lower and upper limits of GFP-AR expression in all subsequent experiments. This analysis enables the selection of HeLa GFP-AR cells with AR expression levels similar to that endogenously expressed in LNCaP cell line.

To further characterize the cell lines, we analyzed them at the single cell level using fluorescence microscopy, and in each line, >90% of the cells were GFP positive. In the absence of ligand, GFP-AR was diffusely distributed cell-wide (Fig. 1B); GFP-AR T877A and GFP-AR F764L were predominantly located in the cytoplasm (Fig. 1C and 1D) in >95% of cells. Upon addition of the synthetic androgen R1881 (10 nM or 100 nM) for two hours, 95–97% had a majority of the signal in the nucleus (Fig. 1B–D). Despite single cell cloning, expression between single HeLa cells was heterogeneous and varied up to 12-fold. Therefore, we used immunofluorescence to determine the relative AR expression level in both stably transfected HeLa and LNCaP cells (Fig. 2A–D) to define a sub-population of HeLa expressing AR at levels similar to LNCaP (Fig. 2E). In subsequent analyses, only this refined, homogenous subpopulation of HeLa cells were analyzed to limit potential over-expression artifacts [3], [16].

**Figure 3:**
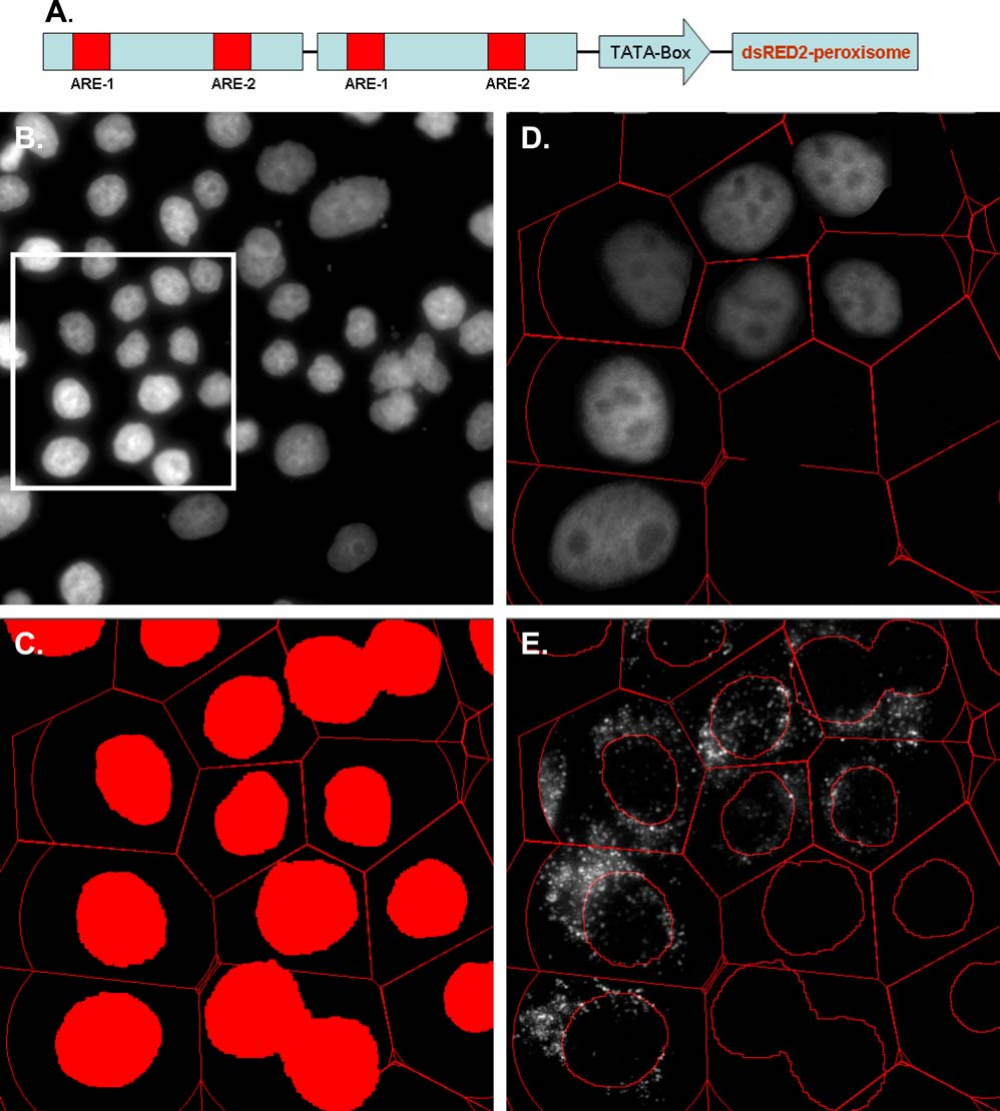
Multiple×assay automated image analysis. A. The probasin proximal promoter element containing two AR binding sites duplicated and fused to a peroxisomes-targeted dsRED2skl protein reporter. B. Raw gray-scale image of HeLa cell nuclei stained with DAPI. White box indicates view used in rest of figure. C. Binary nuclear mask generated by non-linear least squares image filter and image field segmentation based on nuclear centroids and veronoi tessellation. This tessellation in combination with a user defined radius rule defines cytoplasmic compartment of each cell. D. Virtual pseudo color well image generated after GFP-AR data extraction. E. AR transcriptional reporter gene activity at single cell level determined by dsRED2skl image data extraction. All screen captures directly from Cytoshop with various features toggled on/off.

To allow visualization of AR regulated transcriptional reporter gene activity, the HeLa cell lines were transiently transfected with the pARR_2_PB-dsRED2skl reporter construct, based on the AR-responsive composite probasin promoter (Fig. 3A), and then incubated for 18 hours with a 10-point titration (10^−5^ to 10^−14^ M) of the compounds of interest. The dsRED2skl gene encodes a red fluorescent protein that is targeted to the peroxisomes, which improves detection due to concentrating dsRED2skl in the small cytoplasmic organelles. When examining potential anti-androgenic activity, test compounds were titred against 10 nM R1881, a minimal dose found to be sufficient to generate a response in all key measurements. Cells were imaged using an automated microscope with a 40×/0.90 NA objective. For each field, three images were captured: DAPI (nuclei, blue), GFP (AR, green), and dsRED2skl (reporter protein, red). Cytoshop (Beckman Coulter) or Pipeline Pilot (Accelrys) image analysis software was used to identify individual cells in each image. For each cell, the DAPI channel (Fig. 3B) was used to identify the nucleus, and the remaining field was computationally segmented to determine the cytoplasmic compartment for each cell (Fig. 3B–C). These masks were then applied to the green and red images to determine cellular distribution of GFP-AR and transcriptional reporter gene activity (Fig. 3D–E). Cell populations were then sorted to remove nuclei clusters (bi- or multinucleate), abnormal nuclear shape and/or DNA density (apoptotic, mitotic), and to select for low expression levels as defined above. To analyze the GFP-AR subcellular trafficking and transcription results, three key features were determined for each cell: 1) degree of nuclear translocation (**f**raction of GFP signal **l**ocalized **i**n **n**ucleus, FLIN):
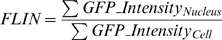
2) amount of nuclear hyperspeckling (**n**uclear **var**iation of GFP signal intensity, NVAR):
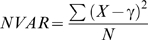
(where X is each nuclear GFP pixel intensity, γ is the average GFP pixel intensity, and N is the number of pixels in the nucleus) and, 3) transcriptional reporter gene activity (total amount of **corr**elated channel **2**/dsRED2skl signal, CORR2):
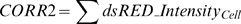
All measurements are then normalized to those observed in untreated and treated (100 nM R1881) GFP-AR. The ability to measure the hyperspeckled patterning is important because it is thought to represent the formation of transient protein complexes by the receptor as it scans the DNA for androgen response elements [22], [23]. The ability of the IC-100 to rapidly focus using a high NA 40× objective was particularly important for these measurements.

### Assay Quality and Repeatability

To determine the repeatability of the minimum and maximum responses (dynamic range) of the assay, we randomly selected and measured 50 cells treated with either the positive control (100 nM R1881, 2 hrs) or the negative control (no ligand). We then calculated the mean, standard deviation (SD), and coefficient of variation (CV) for each measurement for all cells in each of the two wells (Supplementary Table S2). This analysis was repeated 5 times for 5 other pairs of wells. The mean values for each of the three parameters (FLIN, NVAR, and CORR2) did not vary by more than 5% demonstrating that each of these measures is repeatable.

To determine overall assay repeatability, three replicate plates were prepared containing replicates of a 10-point R1881 titration curve with concentrations ranging from 1000 nM to 0.01 nM, and a control well with no ligand treatment. Each plate was imaged once and all measurements were determined (Supplementary Fig. S1A–C). The Z' values, a dimensionless measurement of assay quality based on sample means and their standard deviations, were calculated [24], [25]. A Z' value of 1 is the theoretical “perfect” assay and values between 0.2 and 0.6 are typical for cell based assays [25]. For the replicate sets, the maximum calculated Z' values were 0.76, 0.91, and 0.59 for FLIN, NVAR, and CORR2 and ranged between 0.46 and 0.91 depending on groups and measurements being compared (Supplementary Table S3). Variability in EC50 values calculated upon curve fitting using SigmaPlot software ranged between 5 and 24% (Supplementary Table S4). Reports of EC50 variability in traditional transcriptional reporter gene-based assays have been reported to range between 22 and 57% [26], indicating our HTM-based data is improved in this regard. Since total assay throughput is limited by image acquisition speed, we utilized a derivation of Devore [27] to estimate the minimum number of cell measurements needed per well to achieve significance (Methods). In general, the number of frames per well was set to capture twice the minimum number of cells predicted as necessary to account for any well-to-well differences in the actual number of cells analyzed.

### AR Nuclear Translocation and Hyperspeckling are Distinct Responses

**Figure 4:**
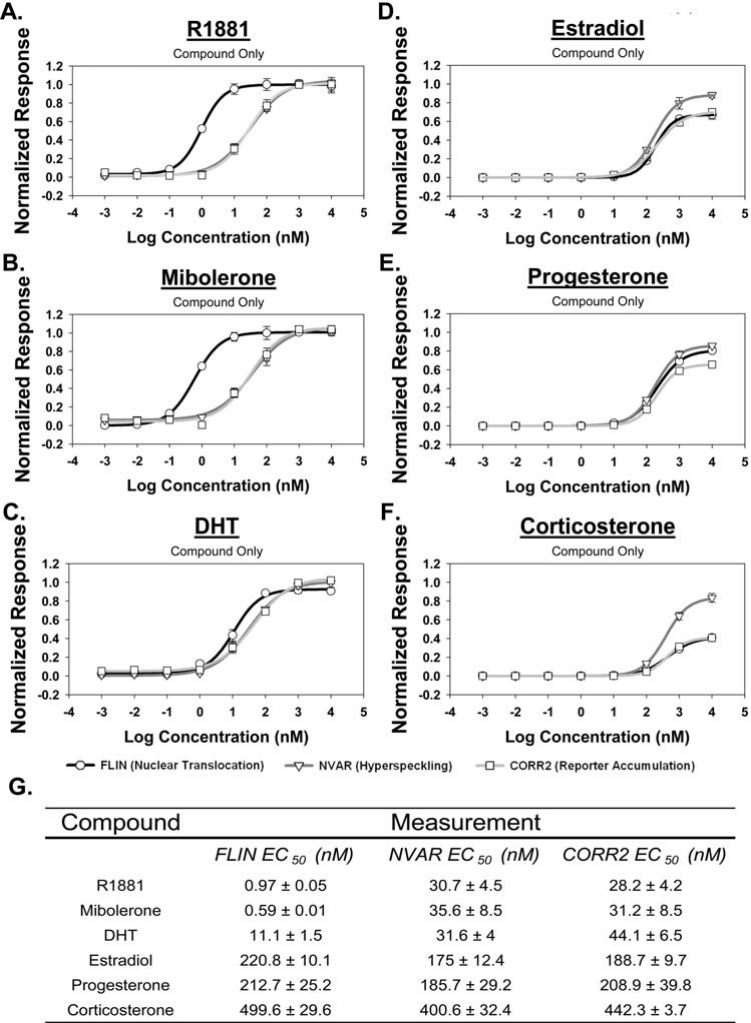
Dose dependent effects measured in panel of steroid compounds. The differential effects of various steroidal compounds on AR nuclear translocation, nuclear hyperspeckling, and transcriptional reporter gene activity in HeLa GFP-AR. Cells transfected with pARR-2PB-dsRED2skl reporter vector and maintained in 5% SD-FBS media for 12 hr. Cells were treated with indicated compound for 18 hr in 5%SD-FBS. Results normalized to negative (no treatment) and positive (R1881) controls. Tested compounds include known AR agonist R1881 (A), mibolerone (B), and DHT(C) as well as other steroidal compounds estradiol (D), progesterone (E) and corticosterone (F). EC50 values calculated using SigmaPlot 4-parameter curve fitting tool and presented±std. error (G). Data represents average of 8 experiments.

To demonstrate the ability to use the assay as a screening tool, known AR agonists that have similar high affinity for AR [28] were tested over a wide range of concentrations, including R1881, mibolerone, and DHT. Whereas DHT can rapidly be metabolized [29], the synthetic androgens R1881 and mibolerone are relatively stable [30]. All three compounds induced GFP-AR nuclear translocation, nuclear hyperspeckling and dsRED2skl transcriptional reporter gene activity in a dose-dependent manner (Fig. 4 and Supplementary Table S6). Using R1881, the calculated EC_50_ concentration for nuclear translocation, nuclear hyperspeckling, and transcriptional reporter gene activity were 0.96±0.03 nM, 30.7±4.5 nM, and 28.2±4.2 nM, respectively. The AR agonists DHT and mibolerone demonstrated similar effects as compared to R1881, differing only in that DHT was approximately 10-fold less efficient in inducing nuclear translocation. It is interesting to note that the EC_50_ for hyperspeckling and transcriptional reporter gene activity were both ∼30-fold higher than that of nuclear translocation, indicating that AR translocation and hyperspeckling/transcriptional reporter gene activity are distinct biological steps and that highly quantitative data can be culled from this multiplex imaging-based approach.

To determine the effects of the GFP fusion on AR function, we directly compared the responses of GFP-AR and untagged AR when transiently-transfected into HeLa cells along with the pARR_2_PB-dsRED2skl reporter gene construct and exposed to various doses of DHT. Both GFP-AR and untagged AR demonstrated similar nuclear translocation responses (Supplementary Fig. S2A) with GFP-AR achieving>90% of the untagged AR response at similar concentrations (EC50_GFP_ = 0.42±0.05 nM, EC50_untagged_ = 2.11±0.50 nM). When the hyperspeckling response is examined (Supplementary Fig. S2B), responses are again at similar concentrations (EC50_GFP_ = 1.21±0.28 nM, EC50_untagged_ = 4.26±0.54 nM), with GFP-AR reaching >55% of the maximal untagged AR response. The transcriptional reporter gene activity response (Supplementary Fig. S2C) is similar to hyperspeckling where GFP-AR achieves ∼50% of the maximal untagged AR response at similar concentrations (EC50_GFP_ = 0.90±0.29 nM, EC50_untagged_ = 1.74±0.24 nM). In all comparisons, the EC50 values were similar between tagged and untagged AR suggesting that the addition of the GFP tag does not significantly interfere with the C-terminal ligand binding domain.

**Figure 5:**
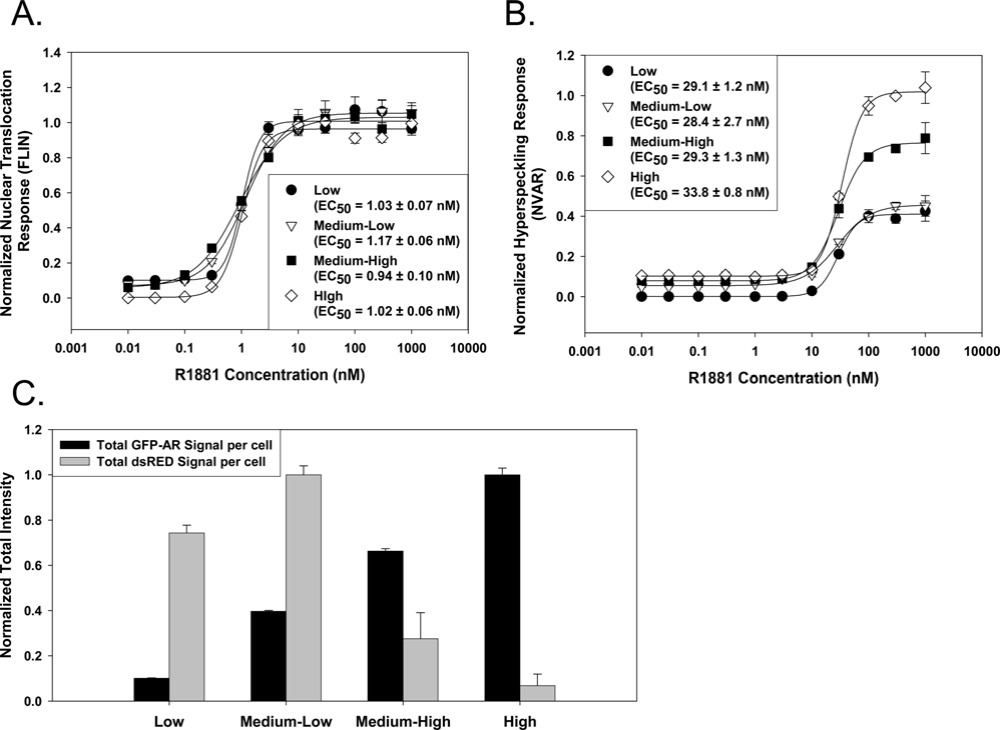
Changes in AR expression level can alter magnitude of responses, but not the concentration at which they occur. To generate a population of cells with a wide range of expression levels, HeLa cells were transiently transfected with GFP-AR and the pARR-2PB-dsRED2skl construct and treated for 18 hr with a R1881 titration. Cells were fixed, DAPI stained, and imaged using the IC100 HTM. Because of image artifacts generated due to the range of expression observed with transient transfections, images were analyzed using Pipeline Pilot software package. After cells were analyzed, the population was subdivided into low, medium low, medium high, and high based on total GFP-AR expression. A. Dose response curves for nuclear translocation failed to demonstrate any affects on the response by AR expression level. B. Dose response curves for nuclear hyperspeckling demonstrated that medium-high and high AR expression was associated with increased amount of hyperspeckling; however, the calculated EC50 values were not significantly different between the populations. C. Analysis of cells treated with 100 nM R1881 demonstrates that transcriptional reporter gene activity in cells with medium-high to high levels of AR expression was significantly (p<0.001) reduced despite increased hyperspeckling.

The ability to perform cell-by-cell analysis allowed us to test the hypothesis that expression level alters the observed responses. At higher levels of expression, an increased magnitude of hyperspeckling was observed with no effect on nuclear translocation (Fig. 5A and 5B). At these higher levels of AR expression (elevated by 2- to 4-fold), transcriptional reporter gene activity was repressed significantly despite the elevated hyperspeckling (Fig. 5C), and completely abolished as expression levels increased. These measurements were taken from cells transiently transfected with GFP-AR in order to poll a range of AR expression beyond that observed in our HeLa stable cell lines. These results suggest that the reason why we are able to achieve high assay quality is largely due to the ability to examine a narrow range of AR expression that is essentially free from potential over-expression artifacts.

In addition to the response observed after 18–24 hrs of ligand treatment, we also quantified responses after a brief pulse of the agonist R1881. Surprisingly, after the HeLa GFP-AR cells were exposed to a 2 hr pulse of 1 nM R1881, the intracellular distribution of AR remained nuclear for up to 15 hr after treatment (Supplementary Fig. S3A). During this time, new protein synthesis was inhibited using cyclohexamide. To ensure the result was not due to residual ligand in the media, we tested the media removed after 15 hrs and were not able to induce the observed response in fresh cells (data not shown). We next reduced the exposure time to either 0.5 hr or 1 hr and again observed nuclear retention of AR (Supplementary Fig. S3A). In contrast, when we examined the nuclear hyperspeckling response after the 2 hr pulse of R1881, the hyperspeckling peaked but then decreased over time (Supplementary Fig. S3A). The nuclear retention is not due to the GFP fusion as we saw similar results when we transiently introduced untagged AR into HeLa cells (Supplementary Fig. S3A).

To determine if part of the nuclear pool of receptor continued to shuttle between the cytoplasm and the nucleus after ligand withdrawal, we used a live cell fluorescence loss in photobleaching (FLIP) assay. The principle of the assay is that if reiterative photobleaching in the cytoplasm results in loss of nuclear fluorescence, the fluorescent protein must be able to shuttle from the nucleus into the cytoplasm. We transfected HeLa GFP-AR cells with a plasmid encoding hcRED (a 25 kDa-red fluorescent protein) to allow visualization of the cytoplasmic region (Supplementary Fig. S3C) and examined cells before ligand treatment and after 5 hours of ligand withdrawal. We observed a rapid loss of nuclear fluorescence with hcRED, consistent with a small protein able to rapidly shuttle between cellular compartments (Supplementary Fig. S3B–C). We also observed a slower, but persistent loss of GFP-AR nuclear intensity in both the untreated, treated, and ligand withdrawn HeLa GFP (Supplementary Fig. S3B–C). The time required for loss of one half of the original nuclear fluorescence was, on average, 15±5 sec (n = 21) for hcRED, 114±18.1 sec (n = 11) for untreated GFP-AR, 612±51.9 sec (n = 11) for treated GFP-AR and 559±43.2 sec (n = 10) for ligand withdrawn GFP-AR. These results are consistent with continued AR shuttling between the nucleus and cytoplasm after ligand withdrawal and with previous heterokaryon assays [31]. These data suggest the presence of post-translational modification(s) and/or long-lasting interactions in the nucleus sufficient to retain AR, also sufficient to cause continued nuclear import of the receptor, but not sufficient to maintain the hyperspeckled pattern. Further, these findings again demonstrate that nuclear translocation and hyperspeckling are distinct biological steps.

We also examined the AR response to other steroid hormones such as estradiol (E2), progesterone (PRO), estrone (EST), corticosterone, and androstenedione. Consistent with previously published cytological and transcriptional results, our single cell analyses revealed that while EC50 values were significantly different, at high concentrations of these steroids, maximal effects rivaled known agonists (Fig. 4, Supplementary Table S6). Interestingly, the ∼30-fold increased sensitivity of agonists for nuclear translocation vs. hyperspeckling and transcription were not observed with these steroids.

### Environmental Anti-Androgens Decrease AR Transcriptional Reporter Gene Activity and Hyperspeckling

**Figure 6:**
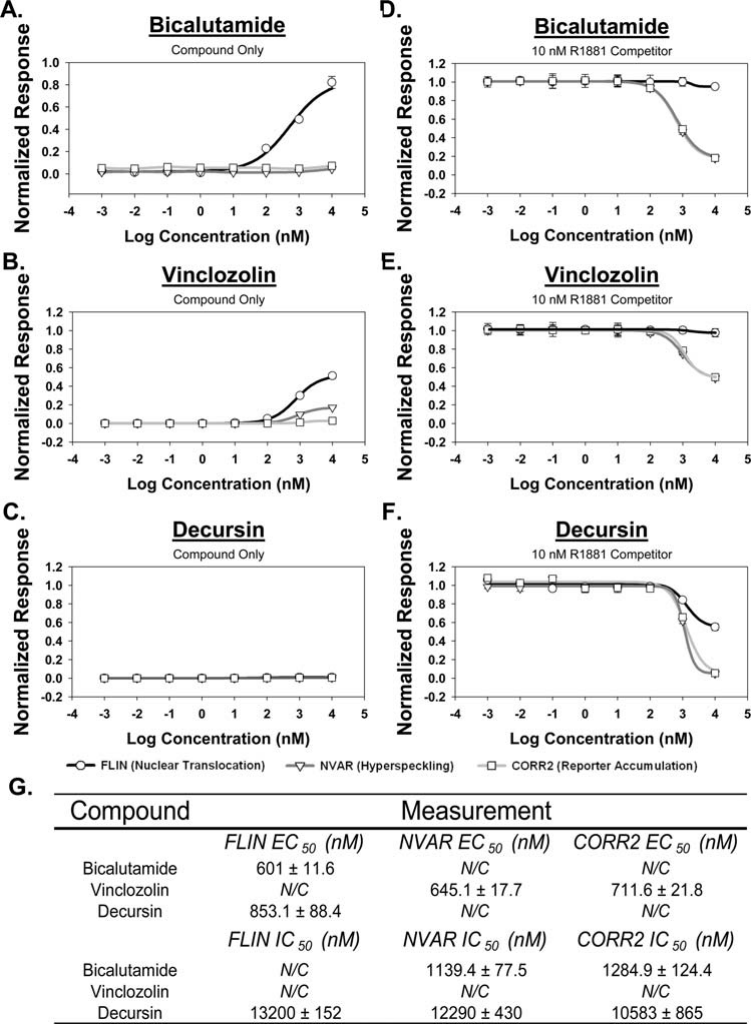
Dose dependent effects measured in panel of AR antagonist compounds. The differential effects of various antagonist compounds on AR nuclear translocation, nuclear hyperspeckling, and transcriptional reporter gene activity in HeLa GFP-AR are plotted. Cells transfected with pARR-2PB-dsRED2skl reporter vector and maintained in 5% SD-FBS media for 12 hr. Cells were treated with indicated compound either alone (A, B, C) or with 10 nM R1881 (D, E, F) for 18 hr in 5%SD-FBS. Results normalized to negative (no treatment) and positive (R1881) controls. When possible, EC50 values were calculated using SigmaPlot 4-parameter curve fitting tool and presented±std. error (G). Data represents average of 8 experiments.

We and others have qualitatively shown that the anti-androgens o-hydroxyflutamide (OHF) and bicalutamide (CAS) can induce AR nuclear translocation, but not the hyperspeckling induced by AR agonists [1]–[3], [32], [33]. To quantitatively confirm these results with our assay system, we tested OHF, CAS and nilutamide (NIL) alone. The three compounds induced nuclear translocation at high concentrations, having EC50 values ∼600-fold higher than R1881 and maximum responses 70%–80% of R1881 (Fig. 6, Supplementary Table S6). As expected for these antagonists, no significant hyperspeckling or induction of transcription was observed.

To characterize the potential antagonistic responses, each test compound was added to the cells approximately 15 minutes before adding 10 nM R1881. As expected, a significant dose-dependent effect on transcriptional reporter gene activity was observed, causing a 71–85% decrease in the dsRED2skl reporter signal (Fig. 6, Supplementary Table S7). Calculated transcriptional repression IC50 values for OHF, CAS and NIL were 677.1±19.2, 645.1±17.1, and 718±31.9 nM. While our calculated IC50 values for CAS after 24 hours are a few fold higher than other cell-based assays (e.g., luciferase) performed at 48–72 hrs, these population-based readouts will also be highly dependent on the actual concentrations of R1881 used for the competition [28]. By examining the other measurements collected, we determined that effects upon transcriptional reporter gene activity were linked to a significant reduction in the ability of AR to develop an agonist-induced hyperspeckled nuclear pattern (81–90%, Fig. 6, Supplementary Table S7).

To further understand the range of responses of AR trafficking and function potential mechanisms, we examined several environmental compounds previously characterized as having anti-androgenic activity. These compounds included Vinclozolin (VNZ; [34]), nitrofen (NF; [2]), and DDT [35], When tested alone, none were able to induce a detectable increase in transcriptional reporter gene activity, despite that fact that all induced modest nuclear translocation of the receptor (45–53% of R1881 response) at high concentrations (EC50 ∼848 to 911 nM). Mechanistically, the lack of a transcriptional reporter gene activity response is also linked to the inability of these compounds to induce a strong hyperspeckling response (Fig. 6, Supplementary Table S6). When incubated with 10 nM R1881, all three compounds reduced transcriptional reporter gene activity (49–55%) with response patterns similar to those observed with CAS, OHF, and NIL (Supplementary Table S7). These results suggest the mechanisms by which these environmental compounds exert their effects may be similar to those observed with the known antagonists OHF, CAS, and NIL.

We also screened a small panel of novel compounds thought to interact with AR. While a majority of these compounds did show a response in terms of transcriptional reporter gene activity (data not shown), the compound Decursin demonstrated a unique response pattern. When observed in combination with 10 nM R1881 (Fig. 6, Supplementary Table S7), the compound caused an antagonistic response (45% decrease in AR transcriptional reporter gene activity, EC_50_ = 13200±152 nM). Surprisingly, however, there was a decrease in R1881-induced nuclear translocation (95%, EC_50_ = 12290±430 nM); further, he small nuclear pool also exhibited a loss of hyperspeckling (94% in NVAR, EC_50_ = 10583±865 nM). These results are consistent with previous biochemical studies and demonstrate the utility of the single cell assay to identify and characterize novel cellular responses based on both cytological and functional readouts [36].

### Response to Additional Compounds

To further explore the utility of our assay, we examined compounds (Atrazine, ATZ; Octophenol, OCT; diethylstilbestrol, DES) that exhibit estrogenic activity, but have rather consistently been reported to lack androgenic activity [28]. These compounds did not yield a significant response on any of the three parameters measured alone or with 10 nM R1881 (Supplementary Table S6 and S7). These results again demonstrated the specificity of the assay and, importantly, suggest that the effects of these compounds are not derived in significant measure from an androgenic or anti-androgenic mechanism.

### Relationship between Cell Cycle and Observed Responses

To emphasize the multiplex nature of this assay further, we examined the relationship between the cell cycle and cellular responses observed with R1881, OHF, and thymidine. To perform these studies, we simplified the HCS cell cycle analysis techniques described by Gesparri et al. [37] to a DNA content (DAPI)/EdU incorporation biparametric analysis to identify G1, S phase, and G2 cells in unsynchronized growing cells (Fig. 6A). Cells were prepared as normal except with a brief 30 minute exposure to 10 µM EdU, which will incorporate into newly synthesized DNA similar to BrdU, prior to fixation. During image analysis, total nuclear DAPI signal (DNA) and mean nuclear EdU signal from each cell was quantified and used to determine cell cycle.

**Figure 7:**
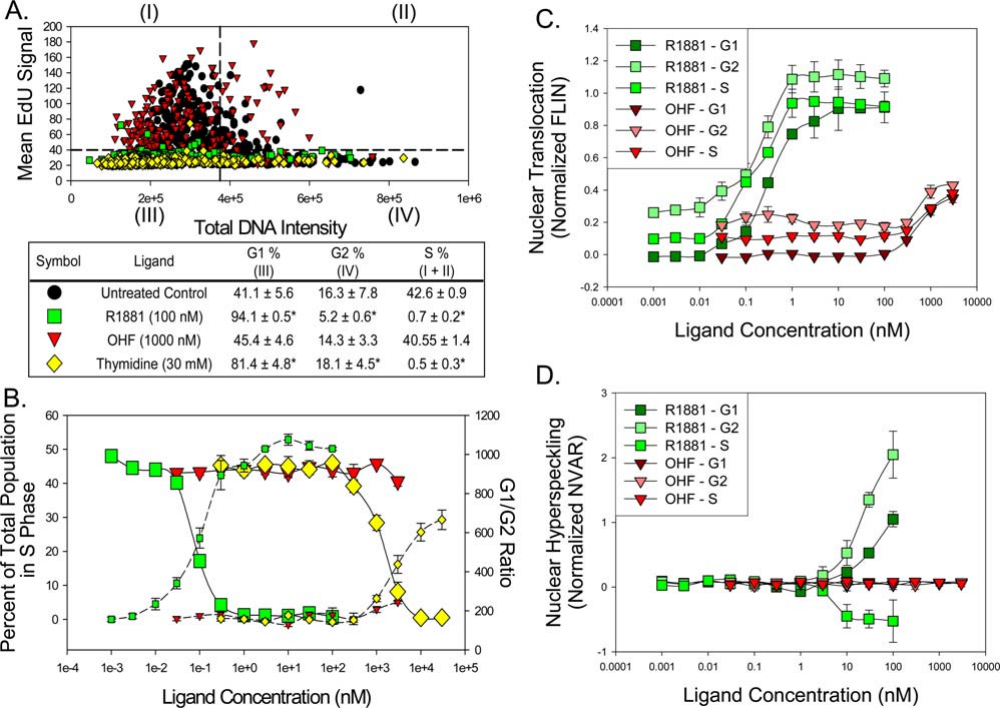
Analysis of the relationship between observed responses, and cell cycle in asynchronous HeLa GFP-AR cells treated with R1881 (green square), o-hydroxyflutamide (red triangle), or thymidine (yellow diamond). A. Biparametric dot-plot analysis of total DNA content (total DAPI signal per nucleus) and 5-ethynyl-2′-deoxyuridine (EdU) incorporation. Dotted lines represent thresholds used to divide cells with either low or high DNA content (vertical line) or cells positively stained for EdU (horizontal line). The percent of cells in each quadrant with each treatment are represented in the associated table. B. Concentration-response curves of the percent of cells in S phase (large markers) and G1/G2 ratio (small markers) after an 18 hr treatment. C and D. Concentration-response curves examining nuclear translocation response (C) and nuclear hyperspeckling (D) in G1, S phase, and G2 cells treated with R1881 and o-hydroxyflutamide. Results normalized to positive and negative controls (all cells).

As expected, thymidine demonstrated a dose-dependent ability to block HeLa GFP-AR cells in G1 (EC50 = 2.6 mM±0.2 mM) and significantly reduced the occurrence of cells in S-phase (42.6%→0.5%, EC50 = 1.30±0.01 mM, p<0.01: Fig. 7A–B). Treatment of cells with R1881 also results in a dose dependent growth arrest of cells characterized by a G1/S block (EC50 = 0.10±0.03 nM) and concomitantly a significant reduction in S-phase cells (42.6%→0.7%, EC50 = 0.07±0.02 nM, p<0.01) (Fig. 7A–B). This response is similar to cell cycle effects of AR when is re-introduced to the PC3 prostate cancer cell line [38]; OHF did not have any appreciable effect on the cell cycle.

By defining the cell cycle position on a cell-by-cell basis, we next determined cell responsiveness to compounds. In untreated cells, G2 cells have a significantly higher percentage of the receptor in the nucleus (data not shown). With an R1881 titration, this pattern persists with G2 cells having 8–12% more AR in the nucleus compared to G1 cells(p<0.05; Fig. 7C). A similar difference is observed with OHF (Fig. 6C). In comparison, there is no significant deference between G1, G2, and S phase cells AR nuclear hyperspeckling in untreated cells (data not shown). In response to R1881, G2 cells have 2.1-fold greater nuclear hyperspeckling response than G1 cells (p<0.01; Fig. 7D). In contrast, S phase cells had a 2.3-fold reduction in the amount of nuclear hyperspeckling compared to G1 cells (p<0.01; Fig. 7D). Cell cycle effects on AR transcriptional reporter gene activity could not be determined due to the reporter accumulation occurring over 18 hrs, during which the cells may continue to progress through the cell cycle. In general, these results show how the AR response is affected by the cell cycle and provide increased accuracy and insight to previous studies examining the relationship between AR transcriptional reporter gene activity and the cell cycle.

### Altered Ligand Responses Observed with T877A (Prostate Cancer) and F764L (AIS) Mutations

Having determined both the agonist and antagonist responses in the assay with wild type AR, we next wanted to examine the assay performance with an AR mutant relevant to prostate cancer. The well-studied T877A mutation, found in the LNCaP cell line, effectively relaxes the stringency of the ligand binding pocket allowing the receptor to become “promiscuous” and respond to a variety of additional ligands [13], [39]. When tested with each compound alone, the effects of the T877A mutation can clearly be seen. Whereas the agonists R1881, mibolerone, and DHT gave results within 1% of that observed with WT-AR, estradiol and progesterone agonist activity increased approximately 300% in all measurements (Supplementary Fig. S4, Supplementary Table S6 and S7). In addition, OHF's potency at inducing nuclear translocation of T877A dramatically increased nearly 100-fold. Further, a 7.5-fold increase in hyperspeckling was observed (EC50 = 125.9±14.1 nM). The same pattern was observed with transcriptional reporter gene activity with a maximal effect increased by 42.5 fold in cells expressing the T877A mutant. These results confirm the agonist response associated with T877A to OHF [13]. The ability to rapidly determine how a mutation affects ligand responses is critical because it is known that the frequency of AR mutations increases in metastatic prostate cancer [40].

**Figure 8:**
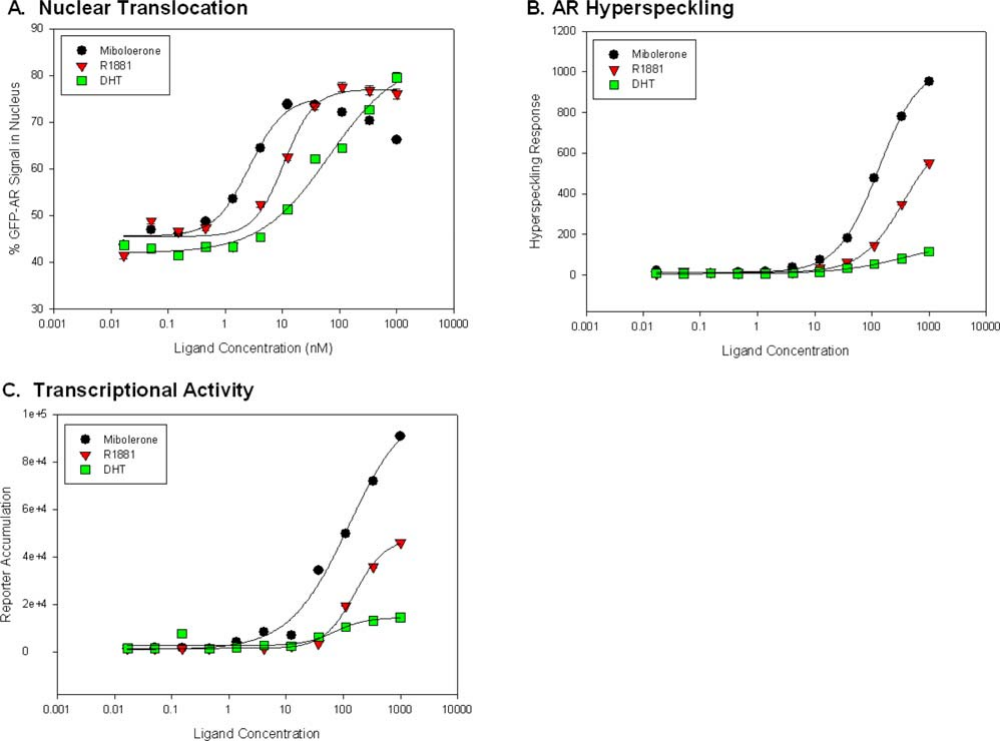
Differential responses of the F764L AR mutation to a panel of agonist. The effects of the F764L mutation on AR nuclear translocation (A), nuclear hyperspeckling (B), and transcriptional reporter gene activity (C) in HeLa cells with treated with R1881 (red), mibolerone (black), or DHT (green). Cells stably expressing F764L form of AR were transfected with pARR-2PB-dsRED2skl reporter vector and maintained in 5% SD-FBS media for 12 hr. Cells were treated with indicated compound for 18 hr in 5%SD-FBS. When possible, EC50 values were calculated using SigmaPlot 4-parameter curve fitting tool and presented±std. error. Data represents average of 4 experiments.

Parallel to studying mutations associated with prostate cancer, we were also interested in characterizing the inactivating mutations associated with androgen insensitivity syndrome. In particular, we sought in compounds that are able to induce a normal response from the mutant receptor. The F764L mutation was isolated from a patient with complete AIS and was previously characterized as having an abnormally high ligand dissociation rate [21]. After generating a HeLa GFP-AR F764L stable cell line, we examined the responses of the mutation when cells were treated with DHT, R1881, and mibolerone at concentrations ranging between 200 nM to 0.02 nM. Consistent with the disease phenotype, DHT failed to induce a strong hyperspeckling or transcriptional reporter gene response even at the highest concentrations tested (Fig. 8 A–C). DHT was able to induce nuclear translocation of the mutant receptor, but only at a high concentration (EC50 = 66.1±7.4 nM). In contrast, when the cells were treated with either R1881 or mibolerone, a marked response was observed in all three parameters examined (Fig. 8 A–C). For R1881, the maximal responses ranged between 40–60% of that observed with the WT AR, but occurred at higher concentrations (EC50 range = 10.6–159.4 nM). Strikingly, higher concentrations of mibolerone were able to induce maximal responses between 85–105% of that observed with the WT receptor (EC50 range = 2.6–71.6 nM). These results not only demonstrate why the disease phenotype is present (e.g., no response to endogenous DHT), but also establishes a rapid and specific ability to identify therapeutically-relevant compounds that may rescue receptor function.

## Discussion

AR develops and maintains the male sexual phenotype under physiologic conditions, and abnormalities in AR function participate in the etiology of several diseases. In the ∼20 years that followed the molecular cloning of AR [41]–[43], biochemical and molecular assays of this molecule have generated a plethora of new information, resulting in an in-depth understanding of AR function, including its involvement in regulating transcription and identification of interacting proteins. Despite these important advances, a simple, rapid, efficient and reproducible model to study mechanisms of AR function remains elusive; numerous separate biochemical techniques are still mandatory to facilitate any hope of interpolation that may provide a systems biology level appreciation of mechanisms. Furthermore, we have not been able to significantly translate any mechanistic advances since AR cloning into improving the fate of patients affected by AIS and ADI prostate cancer. This paper describes the characterization of AR as it becomes transcriptionally activated after addition of agonists, or repressed, after addition of antagonists, using an image based technique that is highly amendable to large-scale screening to identify effectors of AR function, and can serve as a framework to study other nuclear receptors and transcriptional regulators.

The work described here is advantageous over previous cell based assays in several ways and has provided useful insights on AR biology. First, this assay generates multiplex high throughput data that not only measures AR transcriptional reporter gene activity, but also two other upstream steps linked to transcriptional competence (nuclear translocation and hyperspeckling). By having the ability to compare how compounds affect each of the measurements, we were not only able to reiterate that compounds such as R1881 are powerful AR agonists, but also that formation of the hyperspeckled pattern associated with agonist bound nuclear receptors is a distinct mechanistic step from nuclear translocation and occurs only at ∼30-fold higher agonist concentrations. The correlation between formation of the hyperspeckled pattern and transcriptional reporter gene activity lends further support to the concept that this pattern represents an increased trend toward protein complex formation and transcriptional regulation. A similar link between hyperspeckling of other Type I nuclear receptors has been reported (e.g., ER, PR, GR). While the speckles themselves appear to be only randomly associated with sites of transcription, and are demonstrated to be transient in the living cell by photobleaching methods, the degree of AR-AR or AR-coregulator interactions determined by FRET suggest they represent complexes at an ill-defined level of maturation (relevant to activation potential). Alternately, the speckles simply represent the notion that the vast majority of complexes can form away from the very small portion of the nuclear volume that contains target genes. If the latter case were true, an underlying deterministic or stochastic mechanism(s) for AR dynamics would be necessary to facilitate a means for the complexes to find target genes.

We were also able to quantitatively confirm that traditional AR antagonist (OHF, CAS, NIL) are able to induce nuclear translocation fail at forming a hyperspeckled pattern; furthermore, in the presence of agonist, transcription inhibition is linked to blocking the formation of the hyperspeckled pattern. The differences in the amount of speckling observed following exposure to the various compounds used could reflect differences in the identity of the recruited coregulators and/or the strength of AR-CoR binding [44].

We also tested compounds belonging to the rapidly-increasing group known as “endocrine or environmental disruptors,” electing to test agents with known ER or AR antagonistic activity. No induction of AR transcriptional reporter gene activity was detected with our small set of these substances, although both ER (at µM concentration) and AR (at sub µM concentrations) disruptors showed the ability to induce nuclear translocation and some minor hyperspeckling. That these compounds have some affect on two of the three agonist actions (translocation and hyperspeckling) may indicate a mechanistic action unlike the known antagonists. While failing to activate the probasin reporter, the moderate hyperspeckling observed may indicate transcriptional effects on other genes. Panels of AR sensitive transcriptional reporter genes and multi-color mRNA fluorescent in situ hybridization (FISH) experiments are in development to directly test this important question. In the R1881 competition assay, no AR antagonistic activity was detected for the ER disruptors; in contrast, the AR disruptors DDT, Vinclozolin and Nitrofen were effective in preventing hyperspeckling and transcriptional reporter gene activity at concentrations very similar (∼1.5-fold higher) to established AR antagonists (OHF, Casodex and Nilutamide). The presence of these substances in certain environments, and their recognized AR antagonistic activity at concentrations that may be achieved after chronic exposure is important, and according to some investigators may affect AR-mediated functions such as spermatogenesis [45], [46] and testicular development [47]. Utilization of the HTM multiplex AR assay could greatly assist in evaluating potential endocrine disruptor effects on AR functions, cell cycle and toxicity; further, this approach his highly amenable to all other gene regulators, thus greatly expanding the ability to carefully monitor xenobiotic effectors.

The second advantage of our approach is that the microscopic nature of the assay permits the selection of individual healthy interphase cells expressing near endogenous levels of AR. This is an essential methodological characteristic because several critical functions of a nuclear receptor are affected when it is over-expressed, even if only to modestly higher levels [3], [16]. This suggests that bulk population-derived data, where the amount of expressed exogenous protein is not controlled, must be carefully interpreted to avoid confusing normal physiology with pathological expression levels that turn on the cell stress program ([48]; manuscript in preparation). In the work presented here, the ability to closely select the expression level also allowed us to determine that receptor expression levels (within the range examined here) do not markedly affect nuclear translocation or hyperspeckling sensitivity (as determined by EC50 values), but, rather, do markedly affect the magnitude of the hyperspeckling and the transcriptional reporter gene activity responses. The ability to specifically link expression levels to multiple cellular responses will be an important means to examine the functional significance of altered protein expression levels sometimes observed in diseases where AR is moderately over-expressed, such advanced metastatic prostate cancer [49]. Furthermore, as some proteins and/or cells appear to respond variably to over expression levels, access to single cell data linking transcription factor levels to function is fundamental to improved mechanistic understandings.

Along with the ability to correlate responses to expression level, we were also able to link measurements to cell cycle subpopulations. Our results demonstrate that in S-phase and G2 cells, AR is significantly more nuclear than in G1 cells. With agonist treatment, the increased nuclear localization in G2 is associated with heightened nuclear hyperspeckling. In contrast, the S-phase cells demonstrate decreased hyperspeckling with agonist treatment. Although not directly studied here due to technical limitations, these results would predict AR to have the highest transcriptional reporter gene activity in G2 (increased nuclear translocation and hyperspeckling). However, others have found AR to have the highest transcriptional reporter gene activity in G0 and S phase [17]. The causes for this discrepancy could include, but are not limited to, the use of different cell lines, potential artifacts from chemically-based synchronization, or a regional, physical disassociation of hyperspeckling and transcriptional reporter gene activity during DNA synthesis. Utilization of a short-lived fluorescent reporter protein or mRNA FISH of endogenous target genes will be required to bring an improved dynamic readout of gene expression into the multiplex analyses.

Finally, the flexibility of the assay allowed us to examine disease-related AR mutations for altered compound responses. Especially important would be the analysis of AR mutations thought to be involved in prostate cancer treatment resistance [49] or AR mutations involved in the androgen resistance observed in AIS. The observation that hydroxyflutamide acted as an agonist on the T877A mutation is in agreement with previous reports in COS cells [13], and it is significant because this effect was observed at a concentration of 75–85 nM, a readily achieved level in patients treated with this compound. Interestingly, and in agreement with known literature, lower doses of estradiol and progesterone showed the ability to activate the T877A AR mutant. Whether this observation has clinical implications is doubtful. Concentrations of 50–60 nM would still be necessary for these two ligands to activate AR, and these levels are clearly supraphysiologic and not likely achievable in normal individuals, or in patients with prostate cancer receiving hormonal manipulation. For the F764L mutation associated with AIS, it is significant that we were able to observe an increased response with the synthetic androgen R1881 and mibolerone. Because HeLa cells do not express the enzymes necessary to metabolize DHT, the increased responses observed with R1881 and mibolerone are due to characteristics of the ligands, and not higher intracellular concentrations than DHT. The ability to rapidly identify ligands that activate a mutant receptor lends itself to the concept of personalized medicine for AIS patients. To this end, application of our multiplex HTM approach is underway with patient-derived genital skin fibroblasts (Szafran, Mancini and Marcelli, unpublished observations).

Overall, the technology described in this paper represents a significant advance that builds upon our previous efforts to study AR at the single cell level by now allowing a quantitative assessment of multiple aspects of intracellular AR function. The technology is straightforward, reliable, reproducible, and is automated to the point where large libraries of compounds can now be tested to identify novel AR agonists and antagonist, including endocrine disruptors. Moreover, use of ARs harboring patient-related mutations will be amenable to agonist/antagonist screening for personalized patient drug selection. Finally, combined with current RNAi technologies, this multiplex HTM assay should also aid in the identification of proteins involved in pathways that regulate AR biology (Szafran, Marcelli and Mancini, in preparation).

## Methods

### Reagents

All chemicals were obtained from Sigma-Aldrich (St. Lois, MO) unless stated otherwise. Methyltrienolone (R1881) was obtained from NEN Life Science Products (Boston, MA).

### Generation of Stable Cell Lines

Stable HeLa cell lines expressing GFP-AR (wild type and T887A) were generated to ensure GFP-AR protein was expressed in a high percentage of cells. HeLa cells were transfected with 1 µg/well plasmid DNA and 0.01 µg/well linear hygromycin marker (BD) using BioRad Transfectin reagent 1 day after plating in six-well plates. After 24 hours, cells were trypsinized and plated in medium supplemented with 500 µg/ml Hygromycin (Sigma, St Louis, MO) in 10 cm tissue culture dishes. Clones were selected and checked for appropriate GFP-AR distribution and expression by widefield fluorescent microscopy and western blotting. Stable cell lines were maintained in DMEM/F12 medium supplemented with 5%FBS and 500 µg/ml hygromycin.

### High Throughput Microscopy – Sample Preparation

Twenty-four hours before transfection, cells were plated onto 100 mm plastic dishes in medium supplemented with charcoal stripped and dialyzed FBS (SD-FBS). Transient introduction of the pARR-2PB-dsRED2skl reporter construct was performed using 6.0 µg reporter plasmid and 6.0 µg carrier DNA (BlueScript, Stratagene, San Diego, CA) using Transfectin following standard protocols. After 8 hour incubation, DNA/lipid complexes were removed. Cells were then trypsinized and replated at 8500 cells per well in Matrical 384 poly-D-lysine treated 384 well optical glass bottom plates and incubated an additional 12 hours to allow for cell adhesion. Cells were then exposed to for 24 h to ligands at concentrations ranging from 10^−5^ M to 10^−14^ M. Compound dilutions and final addition to multi-well plates were performed using a Beckman Biomek NX robotic platform to ensure repeatability from experiment to experiment. For competition studies, after experimental compounds were added to the cells, the competitor (10 nM R1881) was added to the wells approximately 15 minutes later. After incubation was complete, using the Biomek NX robot, plates were washed with PBS and fixed for 20 min at RT in 4% formaldehyde prepared in CSK buffer (80 mM potassium PIPES, pH 6.8, 5 mM EGTA, 2 mM MgCl_2_). After fixation, cells were briefly permeabilized (5 min) with 0.5% Triton-X and prepared for imaging by washing in PBS, aspirating the washed solution, and adding a 1 ng/ml DAPI solution. Cells were imaged in PBS.

For experiments in which cell cycle effects were determined, cells were labeled using an Invitrogen Click-iT cell cycle analysis kit using supplied protocols. Prior to fixation, cells were exposed to 10 µM EdU for 30 minutes. Cells with EdU incorporation were labeled by an EdU specific antibody conjugated to an A647 florescent marker. Because the Click-iT labeling decreases GFP signal intensity, AR was labeled using an Anti-AR antibody (Dr. Nancy Weigel) and A488 secondary antibody (Molecular Probes).

### High Throughput Microscopy – Imaging

Cells were imaged using the Cell Lab IC-100 Image Cytometer (IC100; Beckman Coulter) platform which consists of 1.) Nikon Eclipse TE2000-U Inverted Microscope (Nikon; Melville, NY) 2.) Chroma 82000 triple band filter set (Chroma; Brattleboro, VT) 3.) An imaging camera: Hamamatsu ORCA-ER Digital CCD camera (Hamamatsu; Bridgewater, NJ) and 4.) A focusing camera: Photoonics COHU Progressive scan camera (Photonics; Oxford, MA). The microscope was equipped with a Nikon S Fluor 40×/0.90NA objective and the imaging camera set to capture 8 bit images at 1×1 binning (1344×1024 pixels; 6.5 µm2 pixel size) with 4 images captured per field (DAPI, GFP/A488, dsRED2skl, A647). In general, 49 images were captured per well for image analysis.

### High Throughput Microscopy – Image Analysis

Images were analyzed using either Cytoshop Version 2.1 (Beckman Coulter) or Pipeline Pilot Version 6.1.5 (Scitegic) analysis software. Nuclear masks were generated by applying a non-linear least-squares image filter combined with automatic histogram based thresholding. General background signal in the GFP channel was corrected by automatic mean background subtraction. Total area of GFP-AR image extraction was determined by intersection of a chosen extraction radius (approximately 25% larger than average nucleus radius) and a Voroni tessellation polygon. Cell populations were filtered to achieve a uniform population of cells without cell aggregates, mitotic cells, apoptotic cells, and cellular debris. Applied gates were based upon 1.) nuclear area 2.) nuclear wiggle (AREA/PERIMETER) 3.) DNA content (DAPI INTENSITY). AR cytoplasm to nuclear translocation, AR nuclear variation/hyperspeckling, and transcriptional reporter gene activity were determined using algorithms within the image analysis software (Fraction Localized In Nucleus (FLIN), Nuclear Variation (NVAR), and total channel 2 intensity (CORR2)). For cell cycle analysis, two additional parameters were collected; total channel 0 nuclear intensity (TOTAL_NUC_DAPI) and average channel 3 nuclear intensity (AVG_NUC_Ch03). All data was exported to Pipeline Pilot and responses were normalized to a 0 to 1 range based on (−) and (+) controls and quadruplicates averaged. EC50 values were calculated by plotting a simple scatter plot of response vs. ligand concentration and using SigmaPlot four parameter logistic curve fitting algorithm. Due to the nature of the curve fitting algorithm, for those responses that did not plateau the response observed at the highest concentration was assumed maximal.

### Live Cell Microscopy - FLIP

For live cell FLIP experiments, HeLa GFP-AR cells grown on 23 mm glass bottom Delta T dishes (Bioptechs), transfected with 0.4 µg of pCMV-hcRED plasmid plus 0.8 µg of carrier DNA using Transfectin (BioRad), and allowed to recovery for 24 hrs before being placed onto a LSM 510 confocal microscope (Carl Zeiss, Thornwood NY) equipped with a 63× (NA 1.4) objective. Cells were maintained at 37°C using a Bioptechs Delta Controller and fresh media containing the appropriate ligand was cycled over the cells. A single Z-section was imaged before and at time intervals following each bleach. The bleach was performed using the laser set 488 nm for GFP at maximum power for 10 iterations (∼1 sec) in a circular region contained within the cytoplasm of the cell. Bleaches were repeated every 10 seconds for the duration of the experiment (700 s) Fluorescent intensities of regions of interest were determined using LSM software and data was exported to Excel (Microsoft, Inc.) to normalize intensity to the pre-bleach image. LSM images were exported as TIF files and final figures were generated using Adobe Photoshop.

### Statistical Analysis

Overall assay quality was determined using the Z' calculation, a dimensionless measurement determined using the following equation:
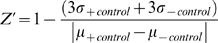
where σ represents the standard deviation of both positive (R1881) and negative control (non-treated) and μ represents the mean of the populations. A Z' value of 1 is the theoretical “perfect” assay and values between 0.2 and 0.6 are typical for cell based assays.

To predict the number of cells needed per well to achieve significant results, we applied a derivation of the Devore equation previously described. Briefly, the Devore equation:
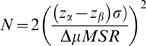
where σ is the overall sample standard deviation, Δμ is the dynamic range of the measurement, and z_α_ and z_β_ relate to type 1 and type 2 errors was used to construct a plot that represents the number of cells needed for the desired minimum significant response. For the assay described here, acceptable type 1 and type 2 errors were set at 0.01 and 0.20 (results presented in Supplementary Table S5).

Determination of significant differences between compounds was accomplished by first performing an ANOVA analysis followed by a post-hoc multiple comparison analysis with significance set at <0.05.

## Supporting Information

Figure S1 Variation between titration curves in the AR agonist, single plate. Three 96 well plates were prepared with HeLa GFP-AR cells transfected with the pARR-2PB reporter construct. Multiple rows were treated with a serial dilution of R1881 ranging from 10−6 M to 10−11 M. In the agonist assay, at low concentrations (far right points) of R1881, (A) nuclear translocation (FLIN), (B) nuclear speckling (NVAR), and (C) AR transcriptional reporter gene activity (CORR2) are minimal. As R1881 concentration increases, a dramatic increase in the measurements is observed with saturation of response observed at ≈100 nM. The color scale represents a range of response from maximal (white) to minimal response (black) for each measurement. All results shown are from a single plate of the set.
(2.38 MB TIF)

Figure S2 GFP-AR responds in a similar manner to untagged AR but with diminished maximal responses. HeLa cells were transiently transfected with either untagged AR or GFP-AR plasmids in addition to the pARR-2PB-dsRED reporter plasmid. Twenty-four hours after transfection, cells were exposed to multiple concentrations of DHT ranging from 0.002 nM to 200 nM for 18 hr. Cells were then fixed and probed with an anti-AR antibody to visualize both GFP-tagged and untagged AR in images captured by the automated IC100 microscope. Images were analyzed using Pipeline Pilot software and the nuclear translocation (A), nuclear hyperspeckling (B), and transcriptional activity (C) responses quantified.
(0.83 MB TIF)

Figure S3 GFP-AR retains a nuclear distribution with decreased hyperspeckling but maintains the ability to shuttle into the cytoplasm after agonist removal. A. HeLa GFP-AR cells were treated with 1 nM for 30 min, 1 hr, or 2 hrs. After ligand treatment, R1881 was removed by serial washes with ligand free media containing cyclohexamide to prevent new protein synthesis. Cells were then fixed, imaged, and examined for the localization of the receptor at 3, 6, 9, 12, and 15 hrs using previously described image analysis tools. Responses were normalized to untreated controls and response seen with 1 nM R1881 treatment for 2 hrs. An additional experiment using untagged AR was also performed to ensure response was not due to the inclusion of the GFP tag on the receptor. The ability of GFP-AR to shuttle between the nuclear and cytoplasmic compartments during and after ligand treatment was analyzed using the FLIP photobleaching technique where a region in the cytoplasm is repeatedly bleached. B. A graph comparing the rate at which nuclear GFP-AR fluorescence is lost in the absence of ligand (untreated, t1/2 = 114±18.1 sec, n = 11), in the presence of 10 nM R1881 (Treated, t1/2 = 612±51.9 sec, n = 11), and after ligand withdrawal (Withdrawal, t1/2 = 559±43.2 sec, n = 10). To ensure results were not due to general photobleaching during imaging, cells were examined where the targeted photobleaching region was outside of the cellular area (Photobleach Control). Both R1881 treatment and withdrawal significantly slow but does not stop the rate that the receptor shuttles between the nucleus and the cytoplasm. C. Selected images from FLIP experiment.
(2.19 MB TIF)

Figure S4 Differential responses of the T877A AR mutation. The differential effects of the T877A mutation on AR nuclear translocation, nuclear hyperspeckling, and transcriptional reporter gene activity in HeLa GFP-AR with selected compounds. Cells stably expressing either WT (unhatched) or T877A (hatched) forms of AR were transfected with pARR-2PB-dsRED2skl reporter vector and maintained in 5% SD-FBS media for 12 hr. Cells were treated with indicated compound either alone (grey bars) or with 10 nM R1881 (white bars) for 18 hr in 5%SD-FBS. Results normalized to negative (no treatment) and positive (R1881) controls. When possible, EC50 values were calculated using SigmaPlot 4-parameter curve fitting tool and presented±std. error. Data represents average of 4 experiments.
(1.51 MB TIF)

Table S1 (0.05 MB PDF)

Table S2 (0.03 MB PDF)

Table S3 (0.04 MB PDF)

Table S4 (0.03 MB PDF)

Table S5 (0.56 MB TIF)

Table S6 (0.06 MB PDF)

Table S7 (0.04 MB PDF)
