# Supplementary figures and images for: Androgen Receptor Functional Analyses by High Throughput Imaging: Determination of Ligand, Cell Cycle, and Mutation-Specific Effects
Source: PLoS One. 2008 Nov 3;3(11):e3605. doi: 10.1371/journal.pone.0003605 (PMC2572143; doi:10.1371/journal.pone.0003605)

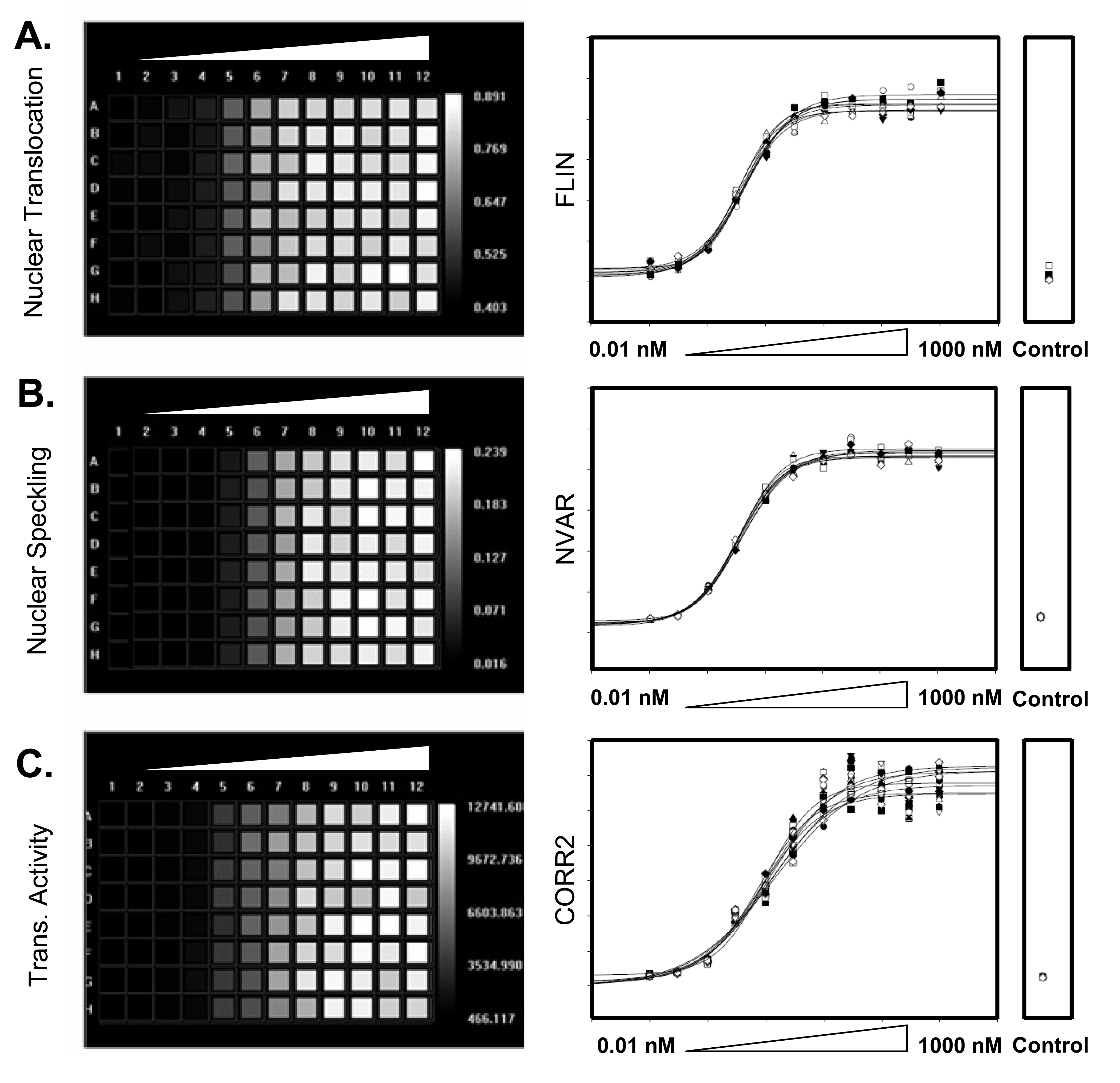

Supplement: Figure S1 — Variation between titration curves in the AR agonist, single plate. Three 96 well plates were prepared with HeLa GFP-AR cells transfected with the pARR-2PB reporter construct. Multiple rows were treated with a serial dilution of R1881 ranging from 10−6 M to 10−11 M. In the agonist assay, at low concentrations (far right points) of R1881, (A) nuclear translocation (FLIN), (B) nuclear speckling (NVAR), and (C) AR transcriptional reporter gene activity (CORR2) are minimal. As R1881 concentration increases, a dramatic increase in the measurements is observed with saturation of response observed at ≈100 nM. The color scale represents a range of response from maximal (white) to minimal response (black) for each measurement. All results shown are from a single plate of the set. (2.38 MB TIF) [file pone.0003605.s001.tif]

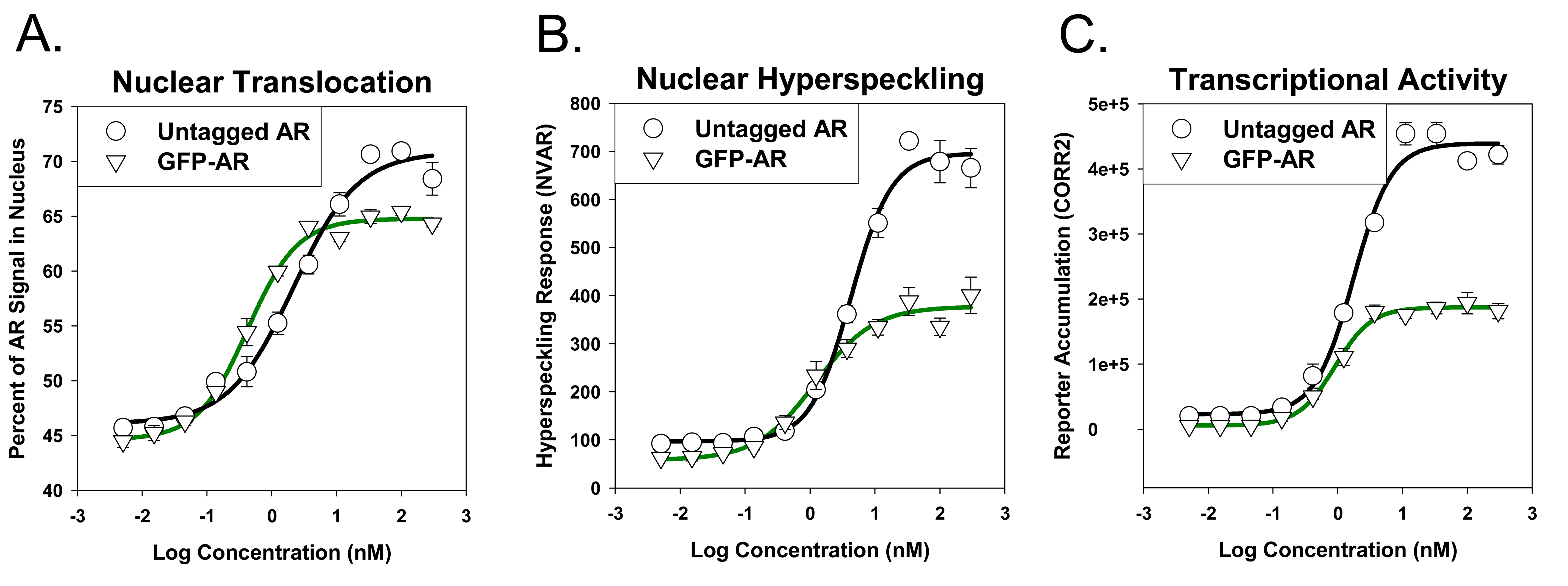

Supplement: Figure S2 — GFP-AR responds in a similar manner to untagged AR but with diminished maximal responses. HeLa cells were transiently transfected with either untagged AR or GFP-AR plasmids in addition to the pARR-2PB-dsRED reporter plasmid. Twenty-four hours after transfection, cells were exposed to multiple concentrations of DHT ranging from 0.002 nM to 200 nM for 18 hr. Cells were then fixed and probed with an anti-AR antibody to visualize both GFP-tagged and untagged AR in images captured by the automated IC100 microscope. Images were analyzed using Pipeline Pilot software and the nuclear translocation (A), nuclear hyperspeckling (B), and transcriptional activity (C) responses quantified. (0.83 MB TIF) [file pone.0003605.s002.tif]

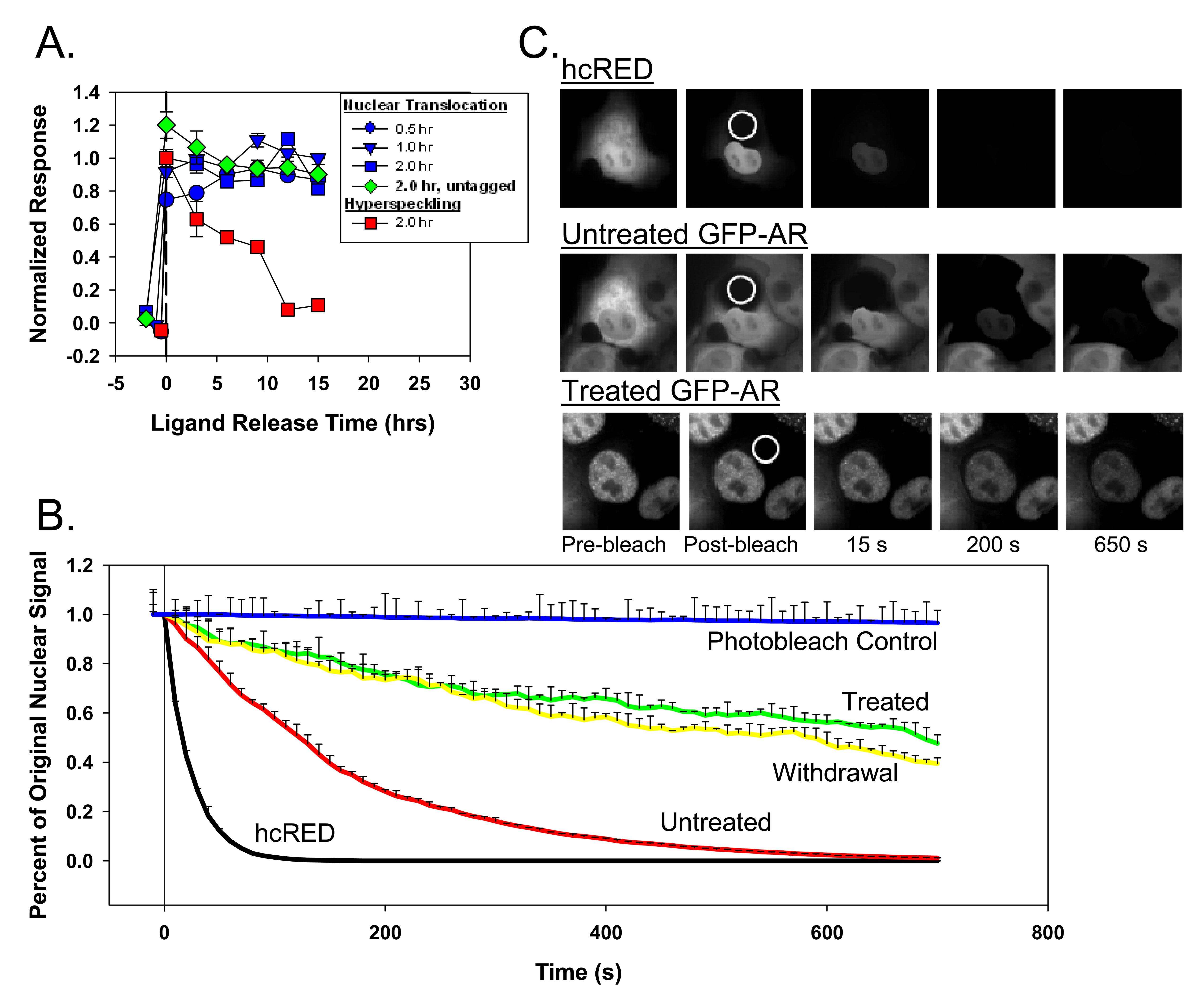

Supplement: Figure S3 — GFP-AR retains a nuclear distribution with decreased hyperspeckling but maintains the ability to shuttle into the cytoplasm after agonist removal. A. HeLa GFP-AR cells were treated with 1 nM for 30 min, 1 hr, or 2 hrs. After ligand treatment, R1881 was removed by serial washes with ligand free media containing cyclohexamide to prevent new protein synthesis. Cells were then fixed, imaged, and examined for the localization of the receptor at 3, 6, 9, 12, and 15 hrs using previously described image analysis tools. Responses were normalized to untreated controls and response seen with 1 nM R1881 treatment for 2 hrs. An additional experiment using untagged AR was also performed to ensure response was not due to the inclusion of the GFP tag on the receptor. The ability of GFP-AR to shuttle between the nuclear and cytoplasmic compartments during and after ligand treatment was analyzed using the FLIP photobleaching technique where a region in the cytoplasm is repeatedly bleached. B. A graph comparing the rate at which nuclear GFP-AR fluorescence is lost in the absence of ligand (untreated, t1/2 = 114±18.1 sec, n = 11), in the presence of 10 nM R1881 (Treated, t1/2 = 612±51.9 sec, n = 11), and after ligand withdrawal (Withdrawal, t1/2 = 559±43.2 sec, n = 10). To ensure results were not due to general photobleaching during imaging, cells were examined where the targeted photobleaching region was outside of the cellular area (Photobleach Control). Both R1881 treatment and withdrawal significantly slow but does not stop the rate that the receptor shuttles between the nucleus and the cytoplasm. C. Selected images from FLIP experiment. (2.19 MB TIF) [file pone.0003605.s003.tif]

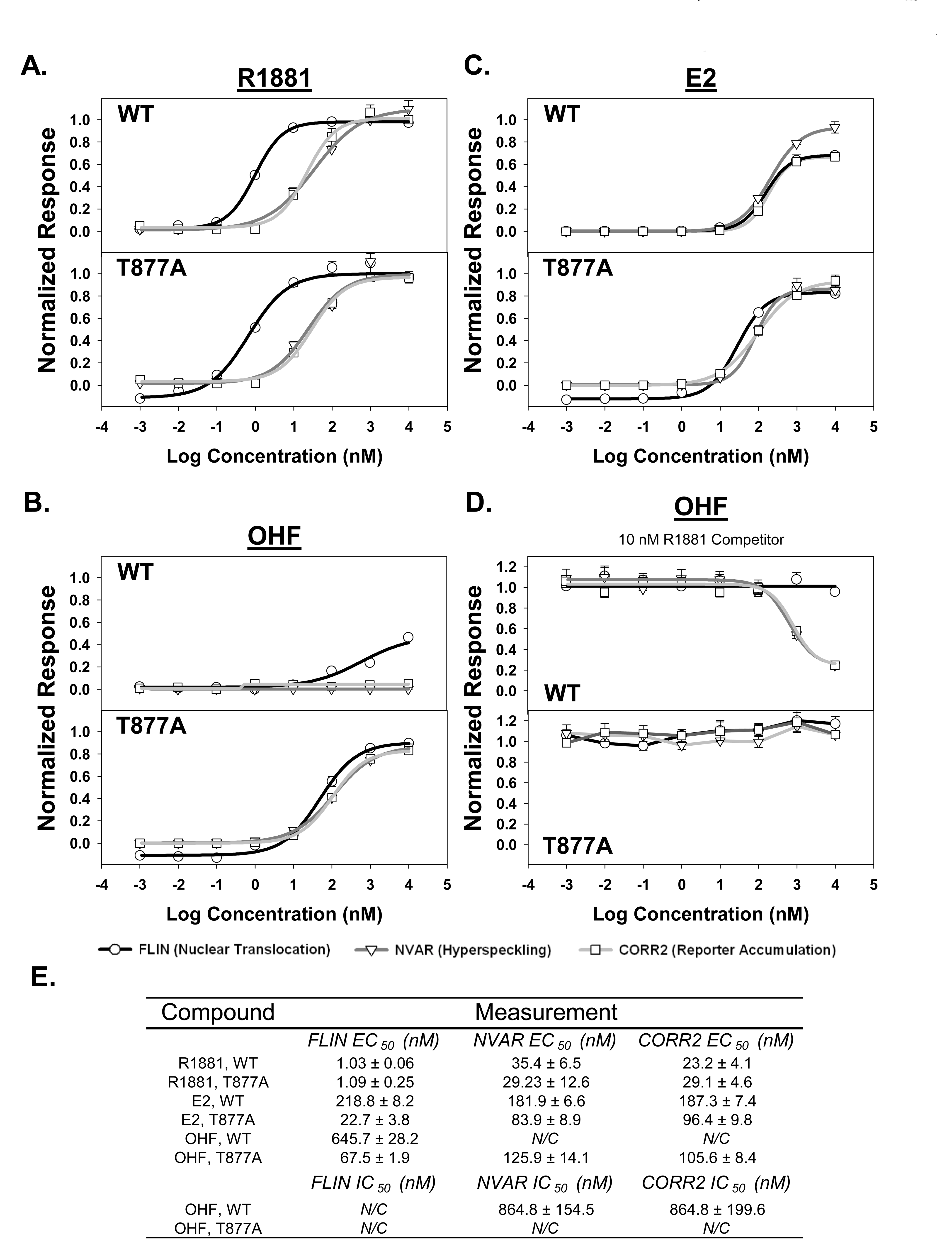

Supplement: Figure S4 — Differential responses of the T877A AR mutation. The differential effects of the T877A mutation on AR nuclear translocation, nuclear hyperspeckling, and transcriptional reporter gene activity in HeLa GFP-AR with selected compounds. Cells stably expressing either WT (unhatched) or T877A (hatched) forms of AR were transfected with pARR-2PB-dsRED2skl reporter vector and maintained in 5% SD-FBS media for 12 hr. Cells were treated with indicated compound either alone (grey bars) or with 10 nM R1881 (white bars) for 18 hr in 5%SD-FBS. Results normalized to negative (no treatment) and positive (R1881) controls. When possible, EC50 values were calculated using SigmaPlot 4-parameter curve fitting tool and presented±std. error. Data represents average of 4 experiments. (1.51 MB TIF) [file pone.0003605.s004.tif]

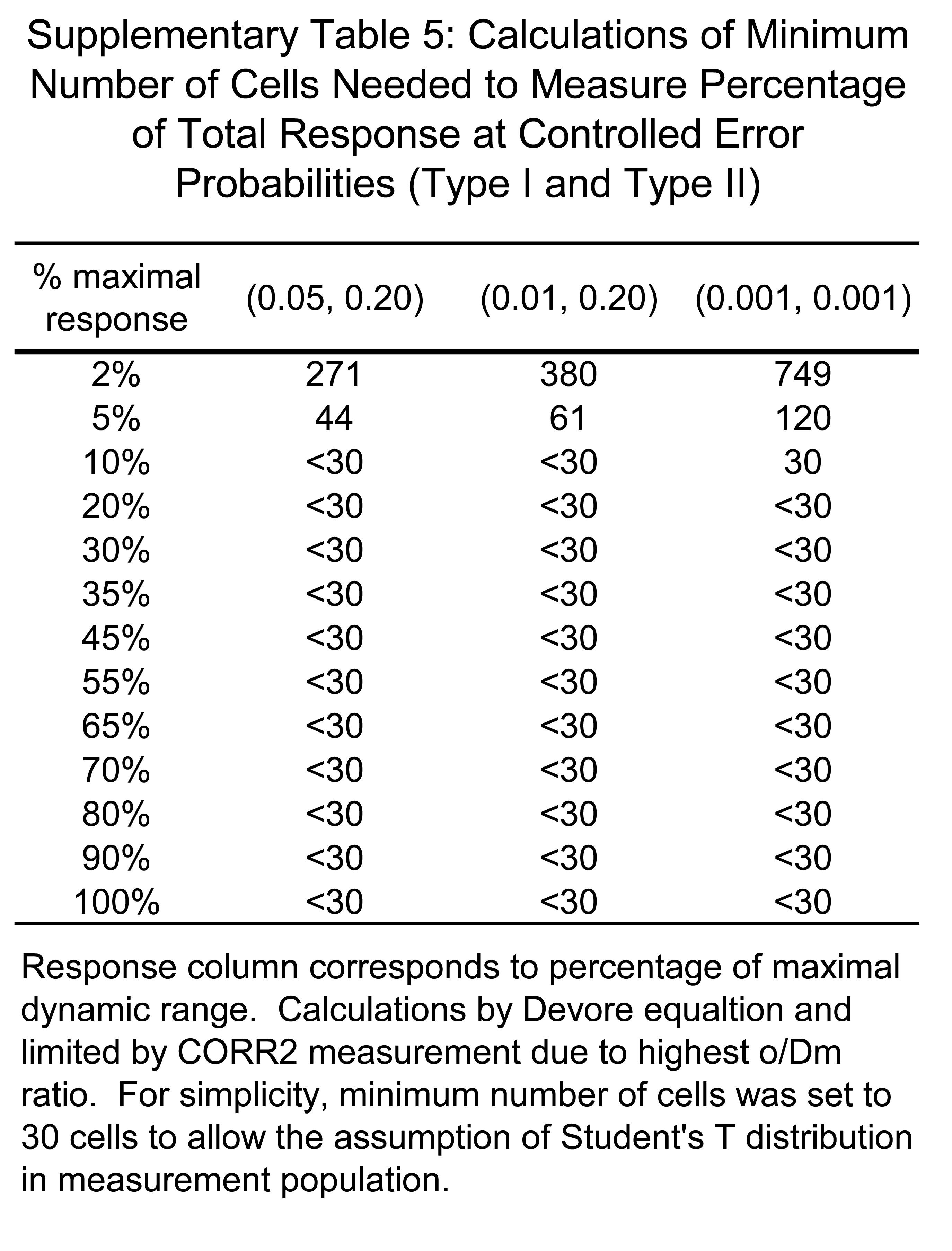

Supplement: Table S5 — (0.56 MB TIF) [file pone.0003605.s009.tif]
