# Supplementary material for: Androgen Receptor Functional Analyses by High Throughput Imaging: Determination of Ligand, Cell Cycle, and Mutation-Specific Effects
Source: PLoS One. 2008 Nov 3;3(11):e3605. doi: 10.1371/journal.pone.0003605 (PMC2572143; doi:10.1371/journal.pone.0003605)
Supplement: Table S1 — (0.05 MB PDF) [file pone.0003605.s005.pdf]

Supplementary Table 1. Gene Expression Analysis of HeLa GFP-AR Cells Treated with DHT

Genes Down-regulated with DHT treatment

***IL6**	FAM84B	IER3	DUSP16	DKFZp727
PHLDA1	GAS1	SLC38A2	HSPA1A	MCART1
DKK1	***GATA2**	NUAK1	USP49	TNFRSF12A
EFNA1	PAPPA	USP36	ACN9	ZNF14
GRAMD3	KRTHA4	STC2	PLEKHG3	***TOB1**
ANKRD1	***FOXA1**	PTPLAD2	BTBD3	***PRKDC**
BHLHB2	RND3	ZBED1	C3orf28	TGFBR3
NR4A2	DACT2	SLC25A25	CITED2	DDIT4
SERTAD4	***CYR61**	ANKRD32	MCM8	TXNRD1
AXUD1	***RGS2**	SERTAD2	C19orf21	LIMA1
ARID5B	STOX2	CTGF	SERTAD1	AVPI1
ZNF331	***TAGLN**	SV2B	LAMA1	ZDHHC11
***ID1**	***MYC**	DUSP1	HSD17B7	C21orf55
C12orf59	SMAD6	***TFAP2A**	DKFZp43	GABAR
ZNF20	ZXDB	STX11	***TPR**	OLFM1
ZNF702	CARF	***CXCR4**	NFIL3	EIF3S10
FAM46A	DUSP2	LRRC1	LOC221955	PIGA
MMP12	OLR1	ANKRD37	LOC58486	RPRM
C8orf4	TSC22D1	***DAB2**	CENPF	GNG11
SLC16A6	BMP2	ZNF223	DUSP4	CCDC14
***ID3**	THBS1	DUSP5	MGC14376	OLFM1
ARL5B	CDC42EP2	C9orf77	IL18	TM4SF1
CDH5	PPP1R15A	PHLDB2	TUFT1	***LDLR**
SNAI2	BAMBI	FAT	USP9X	A2M
EPHA2	ACTG2	***AHR**	STAMBPL1	RABGAP1
SGK	SLC2A3	KRT17	SRRM2	LOC441087
FAM80B	LHX4	***HDAC4**	***TGIF**	HES4

Genes Up-regulated with DHT treatment

CDC20	ZA20D2	HIC2	HBA1
***PGLS**	ST3GAL1	PKP2	PLEKHF1
IFNGR1	FAM105A	MYLIP	KLF15
STOM	CST3	FZD4	INHBB
KCTD3	IRS2	MT1A	***ZFP36**
ODC1	CBX4	MT1X	IGFL1
CDC42EP4	BLM	ZNF189	RASD1
***UCP2**	PDLIM1	SLITL2	***HBB**
***LRP16**	NT5DC3	ERN1	LOC51233
GCHFR	ZFP36L1	UCK1	PGM2L1
MPHOSPH10	HDHD3	ZFX	
STK35	C16orf44	***FOXO1A**	
RHOG	CD59	BCL6	
DYNLL2	ELL2	FOXC1	
OKL38	RHOB	GP1BB	
GADD45A	BAG3	PDK4	
***ELK1**	NRIP1	IRX3	
ATP1A1	PYGB	PTGES	
SLC16A3	TSC22D3	ERRFI1	
S100A10	CITED4	ZNF114	
***CD83**	KIAA1754	FLJ20366	
CAB39	NET1	CDKN1C	
CEBPB	INPP1	LOC126295	
ZNF696	SOX18	RHOU	
CXADR	***PIM1**	SPSB1	
TAF5L	MT2A	BAIAP2	
PPP3CC	SH3TC1	F2RL1	

Gene expression in HeLa GFP-AR stable cell line was performed using Illumina Genome Wide Beadchip analysis with direct hybridization. RNA was isolated from either untreated cells or cells treated with 100 nM DHT for 2 hours. Approximately 8300 expressed genes were identified and of those, 125 transcripts were significantly down-regulated ($p < 0.01$) and 91 transcripts were significantly up-regulated ($p < 0.01$). Of these transcripts, a number have been previously described in literature in association with androgen treatment and are indicated in bold and asterisks. These results suggest that although the HeLa cell line does not endogenous express AR, proteins necessary for AR regulation of gene expression are present.
