# Supplementary material for: Androgen Receptor Functional Analyses by High Throughput Imaging: Determination of Ligand, Cell Cycle, and Mutation-Specific Effects
Source: PLoS One. 2008 Nov 3;3(11):e3605. doi: 10.1371/journal.pone.0003605 (PMC2572143; doi:10.1371/journal.pone.0003605)
Supplement: Table S2 — (0.03 MB PDF) [file pone.0003605.s006.pdf]

Supplementary Table 2. Assesment of Assay Dynamic Range and Measurement Variability

	Mean	SD	CV (%)
Stimulated			
FLIN	0.84	0.04	4.7
NVAR	0.22	0.02	9.1
CORR2	11982	647	5.4
Unstimulated			
FLIN	0.42	0.02	4.8
NVAR	0.015	0.001	6.7
CORR2	587	24.6	4.2

To determine the general quality of the assay, we examined the mininum and maxium response for each measurement (dynamic range) and the varibility associated with each measurement. All measurements where collected using Cytoshop software and based upon the filtered population of cells.
