# Supplementary material for: Androgen Receptor Functional Analyses by High Throughput Imaging: Determination of Ligand, Cell Cycle, and Mutation-Specific Effects
Source: PLoS One. 2008 Nov 3;3(11):e3605. doi: 10.1371/journal.pone.0003605 (PMC2572143; doi:10.1371/journal.pone.0003605)
Supplement: Table S3 — (0.04 MB PDF) [file pone.0003605.s007.pdf]

Supplementary Table 3. Calculated Z'Values Across Replicate Plates For Each Measurement

Z'-Nuclear Translocation (FLIN)

Plate	*Row 1*	*Row 2*	*Row 3*	*Row 4*	*Row 5*	*Row 6*	*Row 7*	*Row 8*	*Combined*	*Z' All*
1	0.74	0.74	0.73	0.75	0.75	0.73	0.75	0.74	0.75	
2	0.76	0.75	0.73	0.74	0.71	0.75	0.73	0.75	0.69	0.72
3	0.63	0.63	0.57	0.63	0.58	0.61	0.59	0.60	0.75	

Z'-Nuclear Hyperspeckling (NVAR)

Plate	*Row 1*	*Row 2*	*Row 3*	*Row 4*	*Row 5*	*Row 6*	*Row 7*	*Row 8*	*Combined*	*Z' All*
1	0.82	0.81	0.83	0.84	0.81	0.82	0.84	0.85	0.85	
2	0.78	0.77	0.79	0.78	0.78	0.79	0.77	0.80	0.79	0.85
3	0.91	0.86	0.84	0.85	0.72	0.77	0.83	0.88	0.87	

Z'-AR Transcriptional Activity (CORR2)

Plate	*Row 1*	*Row 2*	*Row 3*	*Row 4*	*Row 5*	*Row 6*	*Row 7*	*Row 8*	*Combined*	*Z' All*
1	0.46	0.54	0.55	0.52	0.48	0.52	0.50	0.55	0.74	
2	0.74	0.70	0.69	0.73	0.74	0.72	0.73	0.71	0.73	0.74
3	0.68	0.65	0.59	0.67	0.68	0.70	0.70	0.61	0.70	

To determine overall assay quality, Z' values were calculated from three replicate plates containing a R1881 titration on each row. For each row, wells with the minimal (untreated) and maximal response were determined and used to determine the Z'value for the row. To generate a "Combined" Z'value, all data points used to calculate the "Row" Z' values were combined, To generate the "All" Z' 'values, all data points from the three plates were combined.
