# Supplementary material for: Androgen Receptor Functional Analyses by High Throughput Imaging: Determination of Ligand, Cell Cycle, and Mutation-Specific Effects
Source: PLoS One. 2008 Nov 3;3(11):e3605. doi: 10.1371/journal.pone.0003605 (PMC2572143; doi:10.1371/journal.pone.0003605)
Supplement: Table S4 — (0.03 MB PDF) [file pone.0003605.s008.pdf]

Supplementary Table 4. 50% Effective Concentration (EC50) Row to Row and Plate to Plate Varibility

Nuclear Translocation

Plate			EC_{50} Values from each row								
Number		Mean EC_{50}		A	B	C	D	E	F	G	H
1	Mean	0.98		1.10	1.00	0.96	0.82	1.11	0.89	1.05	0.91
	SD	0.10									
	CV	10.6%									
2	Mean	1.00		1.15	1.01	1.01	0.95	0.87	1.10	0.77	1.15
	SD	0.14									
	CV	13.6%									
3	Mean	1.10		1.33	0.99	1.04	1.18	1.17	0.92	1.14	1.01
	SD	0.13									
	CV	11.8%									
All	Mean	1.03	Mean	1.19	1.00	1.00	0.99	1.05	0.97	0.99	1.03
	SD	0.06	SD	0.12	0.01	0.04	0.18	0.16	0.11	0.20	0.12
	CV	6.1%	CV	9.8%	0.9%	4.2%	18.7%	15.2%	11.3%	19.8%	11.8%

Nuclear Variation

Plate			EC_{50} Values from each row								
Number		Mean EC_{50}		A	B	C	D	E	F	G	H
1	Mean	29.85		26.95265	32.71169	29.24941	28.94946	30.64632	30.10641	33.09734	27.08977
	SD	2.29									
	CV	7.7%									
2	Mean	30.30		27.11268	30.05856	28.9737	27.11268	32.09598	30.05856	34.42446	32.55462
	SD	2.61									
	CV	8.6%									
3	Mean	30.62		28.549216	31.720468	31.617144	31.784052	31.251536	31.728416	26.999356	31.291276
	SD	1.81									
	CV	5.9%									
All	Mean	30.26	Mean	27.54	31.50	29.95	29.28	31.33	30.63	31.51	30.31
	SD	0.39	SD	0.88	1.34	1.45	2.35	0.73	0.95	3.96	2.86
	CV	1.3%	CV	3.2%	4.3%	4.9%	8.0%	2.3%	3.1%	12.6%	9.4%

AR Transcriptional Activity

Plate			EC_{50} Values from each row								
Number		Mean EC_{50}		A	B	C	D	E	F	G	H
1	Mean	27.93		29.912624	26.360304	27.535704	32.877244	22.222896	35.747832	21.00792	27.777356
	SD	4.97									
	CV	17.8%									
2	Mean	29.75		32.9056	27.952288	28.080416	35.57008	28.630784	23.263968	34.06512	27.492192
	SD	4.09									
	CV	13.7%									
3	Mean	28.99		35.207073	30.291921	28.833453	29.008584	23.496264	34.397451	27.911862	22.775643
	SD	4.47									
	CV	15.4%									
All	Mean	28.89	Mean	32.68	28.20	28.15	32.49	24.78	31.14	27.66	26.02
	SD	0.91	SD	2.65	1.98	0.65	3.30	3.39	6.85	6.53	2.81
	CV	3.2%	CV	8.1%	7.0%	2.3%	10.2%	13.7%	22.0%	23.6%	10.8%

The EC50 values were calculated for a 12 point titration curve in each row using SigmaPlot. The means, SD, and CV's were calaculated for each row and each plate from single imaging run.
