# Supplementary material for: Androgen Receptor Functional Analyses by High Throughput Imaging: Determination of Ligand, Cell Cycle, and Mutation-Specific Effects
Source: PLoS One. 2008 Nov 3;3(11):e3605. doi: 10.1371/journal.pone.0003605 (PMC2572143; doi:10.1371/journal.pone.0003605)
Supplement: Table S6 — (0.06 MB PDF) [file pone.0003605.s010.pdf]

Supplementary Table 6

Supplementary Table 6. Calculated Relative Maximal Effect and EC50 Values for Wild Type and T877A Androgen Receptor

A	*Wild Type AR (Q22)*			*AR T877A Mutant*		
Compound	Relative Max Effect*	EC50 (nM)	Relative EC50**	Relative Max Effect*	EC50 (nM)	Relative EC50**
			Nuclear Translocation			
Known AR Agonist						
R1881	1.00	1.0	1.000	1.03	1.09	1.000
Mibolerone	1.02	0.6	1.644	1.01	0.60	1.658
DHT	0.98	11.1	0.087	0.99	10.10	0.089
Weak Agonist						
Estradiol	0.70	220.8	0.004	0.85	22.70	0.020
Progesterone	0.81	212.7	0.005	0.86	60.24	0.016
Corticosterone	0.42	499.6	0.002	0.65	310.20	0.003
Estrone	0.45	512.0	0.002	0.55	485.02	0.002
MPA	0.55	645.2	0.002	0.56	550.32	0.002
Androstenedione	0.48	500.0	0.002	0.48	305.98	0.003
Antagonist						
o-HF	0.51	640.1	0.002	0.91	81.25	0.012
Bicalutamide	0.89	601.0	0.002	0.90	602.19	0.002
Nilutamide	0.67	877.7	0.001	0.67	888.60	0.001
Other						
DES	0.25	1200.2	0.001	0.25	1208.09	0.001
Atrazine	0.01	*N/C*	*N/C*	0.01	*N/C*	*N/C*
Octylphenol	0.07	*N/C*	*N/C*	0.07	*N/C*	*N/C*
DTT	0.45	911.1	0.001	0.45	912.34	0.001
Vinclozolin	0.55	853.1	0.001	0.54	848.70	0.001
Nitrofen	0.53	848.7	0.001	0.54	861.20	0.001
Decursin	0.01	*N/C*	*N/C*	*N/T*	*N/T*	*N/T*
			Hyperspeckling			
Known AR Agonist						
R1881	1.00	30.7	1.000	1.00	29.23	1.000
Mibolerone	1.02	35.6	0.862	1.03	35.33	0.893
DHT	0.98	31.6	0.972	1.00	32.18	0.981
Weak Agonist						
Estradiol	0.88	175.0	0.175	0.88	83.90	0.801
Progesterone	0.85	185.7	0.165	0.85	107.11	0.670
Corticosterone	0.84	400.6	0.077	0.83	410.20	0.130
Estrone	0.67	428.8	0.072	0.68	555.02	0.081
MPA	0.73	522.5	0.059	0.74	570.32	0.073
Androstenedione	0.71	401.1	0.077	0.73	395.98	0.130
Antagonist						
o-HF	0.07	*N/C*	*N/C*	0.85	75.45	0.418
Bicalutamide	0.08	*N/C*	*N/C*	0.05	*N/C*	*N/C*
Nilutamide	0.06	*N/C*	*N/C*	0.07	*N/C*	*N/C*
Other						
DES	0.15	1214.6	0.025	0.15	1210.20	0.026
Atrazine	0.02	*N/C*	*N/C*	0.03	*N/C*	*N/C*
Octylphenol	0.06	*N/C*	*N/C*	0.08	*N/C*	*N/C*
DTT	0.21	749.7	0.041	0.21	766.32	0.041
Vinclozolin	0.18	894.5	0.034	0.18	902.17	0.035
Nitrofen	0.10	1496.5	0.021	0.10	*N/C*	*N/C*
Decursin	0.02	*N/C*	*N/C*	*N/T*	*N/T*	*N/T*
			AR Transcripoitonal Acitivity			
Known AR Agonist						
R1881	1.00	28.20	1.000	1.02	29.10	1.000
Mibolerone	0.99	31.20	0.904	1.00	31.89	0.904
DHT	0.95	44.10	0.639	0.98	43.72	0.660
Weak Agonist						
Estradiol	0.67	188.20	0.150	0.94	96.42	0.564
Progesterone	0.65	208.90	0.135	0.65	100.71	0.475
Corticosterone	0.41	442.30	0.064	0.42	429.73	0.093
Estrone	0.44	479.56	0.059	0.44	552.59	0.059
MPA	0.53	522.54	0.054	0.54	588.48	0.052
Androstenedione	0.47	401.08	0.070	0.47	411.24	0.093
Antagonist						
o-HF	0.02	*N/C*	*N/C*	0.87	85.64	0.337
Bicalutamide	0.01	*N/C*	*N/C*	0.02	*N/C*	*N/C*
Nilutamide	0.02	*N/C*	*N/C*	0.04	*N/C*	*N/C*
Other						
DES	0.03	*N/C*	*N/C*	0.03	*N/C*	*N/C*
Atrazine	0.02	*N/C*	*N/C*	0.02	*N/C*	*N/C*
Octylphenol	0.02	*N/C*	*N/C*	0.02	*N/C*	*N/C*
DTT	0.02	*N/C*	*N/C*	0.02	*N/C*	*N/C*
Vinclozolin	0.03	*N/C*	*N/C*	0.03	*N/C*	*N/C*
Nitrofen	0.02	*N/C*	*N/C*	0.02	*N/C*	*N/C*
Decursin	0.02	*N/C*	*N/C*	*N/T*	*N/T*	*N/T*

* As compared to 100 nM R1881

** Calculated by dividing EC50 of R1881 by calculated EC50 of compound

N/C - Unable to acurately fit curve to calculate EC50; N/T - Not tested
