# Supplementary material for: Androgen Receptor Functional Analyses by High Throughput Imaging: Determination of Ligand, Cell Cycle, and Mutation-Specific Effects
Source: PLoS One. 2008 Nov 3;3(11):e3605. doi: 10.1371/journal.pone.0003605 (PMC2572143; doi:10.1371/journal.pone.0003605)
Supplement: Table S7 — (0.04 MB PDF) [file pone.0003605.s011.pdf]

Supplementary Table 7. Calculated Maximal Effect and IC50 Values from Competition Studies, Wild Type and T877A Androgen Receptor

	Wild Type AR (Q22)			***AR T877A Mutant***		
**Compound**	**Relative Max Effect***	**IC50 (nM)**	**Relative IC50****	**Relative Max Effect***	**IC50 (nM)**	**Relative IC50****
			**Nuclear Translocation**			
Antagonist						
o-HF	0.05	*N/C*	*N/C*	0.02	*N/C*	*N/C*
Bicalutamide	0.04	*N/C*	*N/C*	0.06	*N/C*	*N/C*
Nilutamide	0.04	*N/C*	*N/C*	0.04	*N/C*	*N/C*
Other						
DES	0.02	*N/C*	*N/C*	0.00	*N/C*	*N/C*
Atrazine	0.01	*N/C*	*N/C*	0.04	*N/C*	*N/C*
Octylphenol	0.05	*N/C*	*N/C*	0.02	*N/C*	*N/C*
DTT	0.01	*N/C*	*N/C*	0.03	*N/C*	*N/C*
Vinclozolin	0.02	*N/C*	*N/C*	0.01	*N/C*	*N/C*
Nitrofen	0.03	*N/C*	*N/C*	0.02	*N/C*	*N/C*
Decursin	0.45	13200.0	*N/C****	*N/T*	*N/T*	*N/T*
			**Hyperspeckling**			
Antagonist						
o-HF	0.80	677.1	0.95	0.00	*N/C*	*N/C*
Bicalutamide	0.91	645.1	1.00	0.91	649.4	1.00
Nilutamide	0.73	718.0	0.90	0.80	716.6	0.91
Other						
DES	0.00	*N/C*	*N/C*	0.00	*N/C*	*N/C*
Atrazine	0.00	*N/C*	*N/C*	0.00	*N/C*	*N/C*
Octylphenol	0.00	*N/C*	*N/C*	0.00	*N/C*	*N/C*
DTT	0.60	*1225.1*	*0.53*	0.61	*1266.2*	*0.51*
Vinclozolin	0.54	1139.4	0.57	0.54	1124.27	0.58
Nitrofen	0.71	*965.2*	*0.67*	0.73	*1053.3*	*0.62*
Decursin	0.95	12290	0.05	*N/T*	*N/T*	*N/T*
			**AR Transcripoitonal Acitivity**			
Antagonist						
o-HF	0.79	755.0	0.94	0.00	*N/C*	*N/C*
Bicalutamide	0.85	711.6	1.00	0.94	710.0	1.00
Nilutamide	0.75	782.5	0.91	0.88	782.9	0.91
Other						
DES	0.03	*N/C*	*N/C*	0.00	*N/C*	*N/C*
Atrazine	0.02	*N/C*	*N/C*	0.00	*N/C*	*N/C*
Octylphenol	0.02	*N/C*	*N/C*	0.00	*N/C*	*N/C*
DTT	0.55	1334.6	0.53	0.54	1374.9	0.52
Vinclozolin	0.49	1284.9	0.55	0.49	1390.1	0.51
Nitrofen	0.59	1448.90	0.49	0.60	1280.2	0.55
Decursin	0.94	10583	0.07	*N/T*	*N/T*	*N/T*

* As compared to negative control
** Calculated by dividing IC50 of bicaluatmide by calculated IC50 of compound
***Unable to calculate due to no effect by bicalutamide
N/C - Unable to acurately fit curve to calculate IC50; N/T - Not tested
